# Autologous treatment for ALS with implication for broad neuroprotection

**DOI:** 10.1186/s40035-022-00290-5

**Published:** 2022-03-11

**Authors:** Daehwan Kim, Subin Kim, Ashley Sung, Neetika Patel, Nathan Wong, Michael J. Conboy, Irina M. Conboy

**Affiliations:** Department of Bioengineering and QB3 Institute, University of California, Berkeley, CA 94720 USA

**Keywords:** Neurodegeneration, Amyotrophic lateral sclerosis, *SOD1* mutation, Pluripotent stem cells, Secretome

## Abstract

**Background:**

Amyotrophic lateral sclerosis (ALS) is characterized by a progressive loss of motor neurons (MNs), leading to paralysis, respiratory failure and death within 2–5 years of diagnosis. The exact mechanisms of sporadic ALS, which comprises 90% of all cases, remain unknown. In familial ALS, mutations in superoxide dismutase (SOD1) cause 10% of cases.

**Methods:**

ALS patient-derived human-induced pluripotent stem cells (ALS hiPSCs, harboring the SOD1^AV4^ mutation), were differentiated to MNs (ALS-MNs). The neuroprotective effects of conditioned medium (CM) of hESCs (H9), wt hiPSCs (WTC-11) and the ALS iPSCs, on MN apoptosis and viability, formation and maintenance of neurites, mitochondrial activity and expression of inflammatory genes, were examined. For *in vivo* studies, 200 μl of CM from the ALS iPSCs (CS07 and CS053) was injected subcutaneously into the ALS model mice (transgenic for the human SOD1^G93A^ mutation). Animal agility and strength, muscle innervation and mass, neurological score, onset of paralysis and lifespan of the ALS mice were assayed. After observing significant disease-modifying effects, the CM was characterized biochemically by fractionation, comparative proteomics, and epigenetic screens for the dependence on pluripotency. CM of fibroblasts that were differentiated from the wt hiPSCs lacked any neuroprotective activity and was used as a negative control throughout the studies.

**Results:**

The secretome of PSCs including the ALS patient iPSCs was neuroprotective in the H_2_O_2_ model. In the model with pathogenic *SOD1* mutation, ALS iPSC-CM attenuated all examined hallmarks of ALS pathology, rescued human ALS-MNs from denervation and death, restored mitochondrial health, and reduced the expression of inflammatory genes. The ALS iPSC-CM also improved neuro-muscular health and function, and delayed paralysis and morbidity in ALS mice. Compared side by side, cyclosporine (CsA), a mitochondrial membrane blocker that prevents the leakage of mitochondrial DNA, failed to avert the death of ALS-MNs, although CsA and ALS iPSC-CM equally stabilized MN mitochondria and attenuated inflammatory genes. Biochemical characterization, comparative proteomics, and epigenetic screen all suggested that it was the interactome of several key proteins from different fractions of PSC-CM that delivered the multifaceted neuroprotection.

**Conclusions:**

This work introduces and mechanistically characterizes a new biologic for treating ALS and other complex neurodegenerative diseases.

**Supplementary Information:**

The online version contains supplementary material available at 10.1186/s40035-022-00290-5.

## Background

Neuronal cell death causes a plethora of neurodegenerative diseases, including age-related loss of memory and dementias (such as Alzheimer’s disease), Parkinson’s disease, and amyotrophic lateral sclerosis (ALS). These diseases have in common the increased reactive oxygen species (ROS), mitochondrial dysfunctions, protein misfolding and aggregation, neuroinflammation, progressive loss of specific subtypes of neurons, and so far, the lack of a cure.

ALS is a progressive neurodegenerative disease with rapid onset of paralysis and respiratory failure, and morbidity within 2–5 years after diagnosis [1]. More than 5000 patients in the United States receive a diagnosis of ALS each year [1]. Mutations in superoxide dismutase 1 (*SOD1*), oxidative damage and protein misfolding are involved in both familial and sporadic ALS [2–7]. Transgenic mice with human SOD1^G93A^ (B6SJL-TgN[SOD1-G93A]1Gur) are one of the most widely used models to study ALS as they recapitulate the pathophysiology in humans, such as motor neuron (MN) loss, degradation of neuromuscular junctions, axonal degeneration and limb paralysis [3, 8–11].

Various pluripotent stem cells (PSCs), such as embryonic stem cells (ESCs) and induced pluripotent stem cells (iPSCs), are used in various biomedical fields due to their capacity of unlimited self-renewal and the ability to differentiate into multiple cell types [12]. ALS iPSCs have been established and ALS iPSC-derived MNs show pathological characteristics of this disease: diminished neurite length, protein aggregation, and an enhanced apoptosis [13–16] that is reversible by over-expression of Bcl-2 [17].

Considering the limitations of tissue-specific differentiation and oncogenic side-effects of undifferentiated PSCs, stem cell-derived acellular factors might be a safer alternative to cell transplantation [18]. We previously reported the enhancement of old muscle repair by human ESC (hESC)-derived conditioned medium (CM) (hESC-CM), and the ability of this ESC-CM to absorb/neutralize toxic beta-amyloid [19, 20]. Several other studies focused on CM from mesenchymal and adipose stem cells [21–24], but none have shown meaningful reversal of the multiple, complex, and mutually enforcing pathologies of ALS.

## Materials and methods

### Animals

All animal experimental procedures were performed in accordance with the Guide for Care and Use of Laboratory Animals of the National Institutes of Health, and approved by the Office of Laboratory Animal Care (OLAC), UC Berkeley.

SOD1^G93A^ (B6SJL-Tg(SOD1 * G93A)1Gur/J) transgenic mice were purchased from Jackson Laboratory (No. 002726; Bar Harbor, ME). All mice were randomly assigned to receive ALS iPSC-CM treatment or control CM produced by hESCs differentiated into fibroblasts (dF-CM).

### Culture of human pluripotent stem cells (hPSCs) and collection of CM

hESCs (H9, WiCell Research Institute, Madison, WI) and hiPSC (WTC-11) were obtained through the UC Berkeley Cell Culture Facility. The *SOD1*-mutated familial ALS-iPSCs, CS07 and CS53, were obtained from Cedars-Sinai Medical Center (Los Angeles, CA) (Additional file 1: Table S1). All hPSCs were maintained on plates coated with Vitronectin (Life Technologies, Carlsbad, CA) and cultured in Essential-8 medium (Life Technologies) at 37 °C in a 5% CO_2_ atmosphere. All hPSCs were passaged every 7 days by 0.5 mM EDTA (Life Technologies). For collection of CM, hiPSCs were cultured to 80%–90% of confluence. Then, the cells were washed twice (5 min each) with Opti-MEM (ThermoFisher, Carlsbad, CA) and cultured in Opti-MEM for 24 h and the CM was collected as hESC-CM, hiPSC-CM, and ALS iPSC-CM, as published [20]. The collected CMs were centrifuged at 10,000*g* for 30 min at 4 °C, and filtered through a 0.22-μm syringe filter (Millipore-Sigma, Temecula, CA). CMs from the same groups were pooled (7–10 dishes) and stored in aliquots at − 80 °C before use. To obtain CM from differentiated cells, fibroblasts were derived by first differentiating hESCs and hiPSCs into embryoid bodies in 100-mm Petri dishes in Essential 6 (Life Technologies) at 37 °C in 5% CO_2_ for 5 days, and then into fibroblasts for 7 days, followed by two passages on 0.1% gelatin-coated tissue culture dishes (BD Falcon, San Jose, CA) in Dulbecco's Modified Eagle's Medium (DMEM) containing 10% fetal calf serum (FCS), 1% (*v*/*v*) non-essential amino acid, and 1% (*v*/*v*) penicillin–streptomycin. CM from the fibroblasts was collected and used identically to the hPSC CM.

### Culture of human fibroblast cell line

Human lung fibroblast cells, IMR-90 (CCL-186, ATCC, Manassas, VA), were maintained in DMEM supplemented with 10% FCS and 1% penicillin–streptomycin (Invitrogen, Carlsbad, CA) and maintained in a humid atmosphere at 37 °C containing 5% CO_2_. The cells were grown to 70% confluence and subcultured at 1:5 split when passaging.

### Motor neuron precursor (MNP) and MN differentiation

The process of MNP and MN differentiation was as published [25]. Briefly, hPSCs were detached by 0.5 mM EDTA and split 1:10 on Matrigel-coated plates (1:50 dilution). After 1 day, the Essential 8 was changed to neural-inducing medium containing Neurobasal medium (NBM) plus 3 μM CHIR99021 (Tocris, Tracy, CA), 2 μM LDN-193189 (Tocris) and 3 μM SB431542 (Stemgent, Beltsville, MD). The NBM was composed of DMEM/F12 and Neurobasal medium (ThermoFisher) at 1:1, N2, B27, 0.1 mM ascorbic acid (Millipore-Sigma, Temecula, CA), 1× Glutamax and 1× penicillin/streptomycin (all from Invitrogen, Carlsbad, CA). The inducing medium was maintained for 5 days and changed every other day. On day 5, the cultured cells were split at 1:6 with Accutase and cultured in NBM containing 3 μM CHIR99021, 2 μM SB431542, 0.1 μM retinoic acid (Stemgent) and 0.5 μM purmorphamine (Stemgent). After 7 days, MNPs were generated and expanded with MNP medium, which consisted of NBM supplemented with 3 μM CHIR99021, 2 μM SB431542, 0.1 μM retinoic acid, 0.5 μM purmorphamine and 0.5 mM valproic acid (Stemgent). MNPs were passaged every 7 days by exposure to Accutase and cryopreserved in liquid nitrogen in DMEM/F12, 10% Knockout serum replacement and 10% DMSO. To induce MN differentiation, MNPs were dissociated with Accutase and cultured in suspension in the above NBM with 1 μM retinoic acid and 0.1 μM purmorphamine. The medium was changed every other day. After 7 days, MNPs differentiated into MNs. The MNs were then dissociated with Accutase into single cells and plated onto PLO/laminin-coated plates. The MNs were cultured into mature neurons in NBM plus 1 μM retinoic acid, 0.1 μM Compound E (Calbiochem, San Diego, CA), 10 ng/ml insulin-like growth factor 1, 10 ng/ml glial cell-derived neurotrophic factor (ThermoFisher), and 10 ng/ml brain-derived neurotrophic factor (ThermoFisher).

### Isolation of heparin-binding proteins from CM

The heparin-binding proteins of PSC-CM were prepared as published [20]. Briefly, heparin-agarose type I beads (H 6508, Sigma-Aldrich, St. Louis, MO) were washed with molecular-grade water and incubated with CM from the various cells for 2 h with agitation at 4 °C. Then the samples were centrifuged at 10,000*g* for 20 min. The collected heparin beads were washed twice for 10 min at 4 °C in washing buffer (1 ml phosphate-buffered saline [PBS] + 0.05% Tween-20). The heparin-binding proteins were eluted twice for 15 min at 4 °C in 400 μl of elution buffer (0.01 M Tris–HCl pH 7.5 + 1.5 M NaCl + 0.1% BSA). The collected proteins were re-equilibrated to the cell culture medium by dialysis for 2 h at 4 °C in 500 ml of McCoy's 5A Medium (Gibco, Carlsbad, CA) followed by overnight dialysis at 4 °C in 200 ml of Opti-MEM (Gibco). The eluted heparin-bound proteins were diluted to 800 μl with Opti-MEM and stored at − 80 °C before use.

### Isolation of exosomes from CM

For the isolation of exosomes, 100-ml samples of hESC-CM were centrifuged at 2000*g* for 10 min to remove cells and debris, followed by centrifugation at 10,000*g* for 30 min at 4 °C to remove microvesicles [26]. The supernatant was filtered through a 0.22-μm filter (Millipore-Sigma, Temecula, CA) and then the filtered CM was sedimented by ultra-centrifugation at 120,000*g* for 90 min in an i70 rotor (Beckman Coulter, Brea, CA). The pellet was washed once with PBS and re-sedimented at 120,000*g*, 4 °C for 90 min. Finally, the pellet was resuspended in 200 μl of PBS and stored at − 80 °C for further use.

### Treatment of cyclosporine (CsA)

MNs were cultured with 10 μM CsA (11–011-00, Tocris) for 3 or 5 days. Then, cell viability, apoptosis, real-time polymerase chain reaction (PCR), and enzyme-linked immunosorbent assay (ELISA) were performed.

### Cell viability assay

After treatment with H_2_O_2_ and/or CM or CM-derived factors, the number of viable cells was determined by a resorufin-based assay using the commercially available CellTiter-blue® cell viability assay (G8080, Promega, Sunnyvale, CA), according to the manufacturer’s protocol. MNs were cultured in a 96-well plate at a cell density of 2 × 10^4^ and treated with H_2_O_2_ and/or CMs for 24 h before performing the viability assay. A SpectraMax iD3 Multi‐Mode Microplate Reader (Molecular Devices, Sunnyvale, CA) was used to measure fluorescence intensity (560/590 nm).

### Antioxidant assay

The antioxidant assay was performed according to the manufacturer’s instructions (DTAC-100, BioAssay systems, Hayward, CA). Briefly, each sample (20 μl) was mixed with kit reagent (100 μl). Then the mixed sample was incubated for 10 min at room temperature. The color intensity at 570 nm was measured by a SpectraMax iD3 Multi‐Mode Microplate Reader. The Trolox values ranging 0–1000 μM were used as a reference.

### Real-time PCR

Total RNA was extracted using the RNeasy mini kit (Qiagen, Valencia, CA), and the SuperScript III First‐Strand Synthesis System (Invitrogen, Carlsbad, CA) was used to synthesize cDNA according to the manufacturer’s instructions. Real‐time PCR was performed on a Bio‐Rad iQ5 real‐time PCR machine. The primers used for PCR are listed in Additional file 1: Table S1.

### Immunofluorescence staining

Cells were fixed in 4% paraformaldehyde for 30 min, permeabilized with 0.25% Triton X-100 and blocked with 5% FCS in PBS for 1 h. The fixed cells were incubated overnight at 4 °C in PBS + 1% FCS with antibodies mouse anti-OCT4 (1:500, ab18976, Abcam, Cambridge, MA), rabbit anti-SOX2 (1:500, MA516399, ThermoFisher), rabbit anti-TUJ1 (1:500, MAB1195, R&D system, Minneapolis, MN), rabbit anti-OLIG2 (1:500, NBP128667, Novus), rabbit anti-HB9 (1:500, ABN174, Millipore-Sigma, Temecula, CA), rabbit anti-CHAT (1:500, AB144P, Abcam, Cambridge, MA), and rabbit anti-cleaved caspase-3 (CC3, 1:500, 9669S, Cell Signaling Technology) followed by incubation with secondary antibodies: FITC-conjugated anti-mouse IgG (1:1000, A21202, Life Technologies), FITC-conjugated anti-rabbit IgG (1:1000, A11034, Life Technologies), Cy3-conjugated anti-mouse IgG (1:1000, A11003, Life Technologies), and Cy3-conjugated anti-rabbit IgG (1:1000, A11035, Life Technologies). The treated cells were covered with Slowfade antifade with DAPI (Life Technologies) for nuclear staining and covered with a glass coverslip. Images were captured with a fluorescence microscope (DM5000B, Leica, Wetzlar, Germmany).

### Apoptosis assay

The apoptosis assay of fibroblasts was performed using Annexin V–CF Blue/7-amino-actinomycin D (7-AAD) Apoptosis Detection Kit (ab214663, Abcam) according to the manufacturer’s protocol. Briefly, cells were detached using 0.05% trypsin and washed twice with PBS. Then, samples were resuspended in 1× annexin-binding buffer and incubated with 5 μl of Annexin V-FITC and 5 μl of7-AAD for 15 min at 37 °C, avoiding light. Finally, the stained samples were analyzed on a Guava Easycyte Flowcytometer (Millipore-Sigma, Temecula, CA) at an excitation wavelength of 488 nm and emission filters of 525 and 625 nm. For MNs, the apoptotic assay was conducted on two different markers, FITC-conjugated Annexin V (ab201540) or CC3 (9669S). For Annexin V detection, MNs in 96-well plates were washed twice with PBS. Then, 100 μl of Annexin V binding buffer was added with 5 μl Annexin V-FITC for 5-min incubation at 37 °C, avoiding light. The stained plate was analyzed on a SpectraMax iD3 Multi‐Mode Microplate Reader (490/525 nm). For the CC3, the immunofluorescence protocol was used.

### High-content analysis

For analyses of differentiation efficiency, neurite outgrowth, and apoptosis, the plates were imaged using the high-content imaging system, ImageXpress Micro (IXM, Molecular Devices); a set of 5 × 5 or 7 × 7 fields was collected from each well using the 10× or 20× objective. Data were further analyzed in MetaXpress 6 software (Molecular Devices).

### Analysis of neurite length

During differentiation into MNs, cells were sampled at 5-day intervals between 20 and 60 days. MNs were cultured in 8-well chamber slides (LabTek II CC2-coated), stained with Tuj1 and imaged by IXM. MetaXpress 6 software was used to analyze the mean process length as a measure of total outgrowth divided by the number of processes of a cell. Analysis was performed on a total of 4–10 fields and at least 1000 cells per group from three independent experiments were analyzed. For neurite size comparison, the data were normalized to the values at day 20; shrinkage of neurites over time was determined as the net change from neurite sizes on days 20 and 25.

### ELISA

ELISA was analyzed for IP-10 protein (DIP100, R&D System, Minneapolis, MN) and the sample dilution was performed according to the manufacturer’s protocol (1:20–1:50). The stained plate was analyzed on a SpectraMax iD3 Multi‐Mode Microplate Reader.

### Antibody array

CMs from PSCs or differentiated cells were analyzed on a human L1000 antibody capture array (AAH-BLG-1000, Raybiotech, Norcross, CA), processed according to the manufacturer’s protocol. The array slides were imaged by a Molecular Devices 4000b scanner and data were calculated by Genepix. After normalization to dF-CM, proteins with levels over twofold up- or 0.5-fold down-regulated were selected.

### Epigenetic inhibitor screen

Separate cultures of hiPSCs were incubated for 3 days with one of the following small molecules: 5 μM Nanomycin A (A8191, Apexbio, Walnut, CA), 10 μM LSD1 inhibitor (489476, Millipore-Sigma, Temecula, CA), 5 μM 5-Azacytidine (C832A53, Thomas Scientific, Sanjose, CA), and 10 μM GSK126 (67-905, Tocris, Tracy, CA). After 3 days of treatment, CM was collected (without the inhibitors) using the CM collection protocol as described above.

### Measurement of mitochondrial stress

MNs were detached with Accutase, washed with PBS and incubated for 10 min with 5 μM Mitosox Red (M36008, ThermoFisher) at 37 °C in the dark. After three washes in warm PBS, the stained MNs were resuspended in warm FACS buffer (1% FCS in PBS) and analyzed by a Guava easyCyte flow cytometer (620/52 nm).

### Bioinformatics analysis

The genes of differentially regulated proteins were analyzed using DAVID Bioinformatics Resources (version 6.8, https://david.ncifcrf.gov). The GO (Gene Ontology) functional annotation based on the biological process, molecular function and cellular component, and Kyoto Encyclopedia of Genes and Genomes (KEGG) pathway were performed. Heat maps were generated with ClustVis [27]. Violin plots were constructed with the Python seaborn package and normalized with a standard min–max scaler.

### CM treatment and behavioral test

The CM of ALS iPSCs (CS07 and CS053, Cedar-Sinai) or CM of fibroblasts was injected subcutaneously at the neck in a volume of 200 μl every other day as described [28].

For behavioral test, we monitored the weight, neurological scoring, and hanging time every other day. First, all mice were weighed. Then, we assayed them by neurological scoring, as published [29] with criteria as follows:

- *Score of 0* While mouse is suspended by the tail, the hindlimbs are fully extending away from the lateral midline and the status kept for 2 s.
- *Score of 1* Partial collapse of leg extension or trembling of hind legs during tail suspension. This stage is considered an onset of symptoms.
- *Score of 2* While mouse is suspended by the tail, the hindlimbs are not extending much or completely collapse. During walking of 90 cm, toes curling or dragging of leg parts is observed.
- *Score of 3* Rigid paralysis or minimal joint movement is observed. Hindlimbs not being used much for forwarding motion. We considered this stage the end-stage.

All mice were euthanized at a score of 3. All information was recorded for each mouse every other day until the end-stage (a score of 3).

For hanging test, mice were placed on a grid and then turned upside down. Then, the latency to fall was measured. The latency time measurement began from the point when the mouse was hanging free on the grid and ended with the animal falling to the cage underneath the grid.

### Survival

For survival assessment, 11 female (8 ALS iPSC-CM group and 3 dF-CM group) and 11 male (8 ALS iPSC-CM group and 3 dF-CM group) SOD1^G93A^ mice were used. Animals that showed signs of paralysis with a score of 3 were euthanized, e.g., the non-survival group.

### Tissue collection

When mice showed the first signs of paralysis in hind limbs or at 120 days, euthanasia was proceeded with the guidelines of UC Berkeley’s OLAC administration. For histological comparison, some ALS mice were euthanized at 120 days. Spinal cords and muscles (gastrocnemius and tibialis anterior) were isolated. Then, spinal cords were fixed with 4% paraformaldehyde overnight at 4 °C and muscle tissues were fixed for 30 min at room temperature. Then, all tissues were transferred to 30% sucrose solution overnight and embedded in tissue-tek optimal cutting temperature (OCT, Sakura Finetek, The Netherlands) and snap frozen in isopentane cooled to − 70 °C with dry ice. Then, all samples were stored at − 80 °C before use.

For histological analysis, all OCT-embedded tissues were cryosectioned by a Cryostar NX50 (ThermoFisher). Muscle tissues were sectioned to 20-µm thickness while spinal cords were cut at 10 µm.

### Muscle weight

To compare the wet muscle weight of the gastrocnemius and tibialis anterior in all groups, mice were euthanized and muscle tissues were isolated, and both left and right muscles were weighed on a scale. For comparison, the weight recorded was normalized to the body weight.

### Cresyl violet staining and quantification

Mounted frozen sections were dried overnight at 37 °C and slides placed directly into 1:1 alcohol/chloroform overnight, then rehydrated through 100% alcohol to distilled water. Next, the sections were stained in 0.1% cresyl violet solution for 5–10 min at 37 °C, rinsed with distilled water, dehydrated with ethanol, cleared in xylenes, and mounted in Permount (ThermoFisher). Stained sections were imaged at 5× and 10× magnifications. For quantification, five sections were selected randomly and the total number of MNs of the ventral spinal cord was counted using unbiased methods, as described [30].

### Neuromuscular junction (NMJ) staining and quantification

NMJ analysis was performed as previously described [31]. Briefly, dried sections were permeabilized with 1% Triton X-100 and blocked with 5% FCS in PBS for 1 h. The fixed cells were incubated for 2 h at room temperature in PBS + 1% FCS with Alexa 488-conjugated anti-neurofilament (1:500, 8024, Cell Signaling Technology), Alexa 488-conjugated anti-synaptophysin (1:500, MAB5258A4, Millipore-Sigma, Temecula, CA), or Alexa 555-conjugated anti-α-bungarotoxin (1:500, B35451, Life Technologies). The stained slides were covered with Slowfade antifade with DAPI (Life Technologies) for nuclear staining and covered with a glass coverslip. Images were captured with a fluorescence microscope (DM5000B, Leica). For quantification, five sections were selected randomly and counted. Intact NMJs (yellow) were identified as those that exhibited an overlay of α-bungarotoxin (red) and neurofilament (green). Denervated NMJs were defined by labeling with only α-bungarotoxin (red). Percent of intact NMJs was calculated as the number of intact NMJs (yellow)/total number of motor end plates (yellow + red) as described [31].

### Statistical analysis

All statistical analyses were performed using GraphPad Prism software version 9 (GraphPad software Inc., San Diego, CA). All values are expressed as means ± SEM for independent experiments, or SD for replicates. To determine the significance of differences among groups, comparisons were made using Student’s *t* test. Survival and onset data were analyzed with Kaplan–Meier curves and log rank test. *P* < 0.05 was considered as statistically significant.

## Results

### Autologous ALS iPSC-CM is neuroprotective for MNs of ALS patients

hESC-CM was previously shown to enhance proliferation of myoblasts and rat neural precursor cells [19, 20], but the effects on cell viability were less studied. We decided to explore a protective role of hESC-CM when cells experience oxidative damage, which is physiologically and clinically relevant in a number of human diseases [32, 33]. hESC-CM protected human fibroblasts and human MNs that were differentiated from the hESCs from H_2_O_2_-caused cell death (Additional file 2: Figures S1 and S2). Additional antioxidant activity of hESC-CM was tested and not found (Additional file 2: Figure S3), suggesting that it did not simply neutralize ROS to preserve cell viability. The control dF-CM did not have such neuroprotective properties and was used as a negative control throughout this study.

Encouraged by the neuroprotective effects of hESC-CM on human MNs, we tested the hypothesis that iPSC-CM is also neuroprotective and will promote the viability of ALS patient-derived MNs. If true, this would suggest a novel completely autologous approach to treating neurodegenerative diseases. We studied two ALS patient-derived iPSC lines (CS53-male and CS07-female) with the most common mutation in the *SOD1* gene, A4V (Additional file 1: Table S2). These ALS-iPSC lines were confirmed for their markers of pluripotency and then differentiated into MNPs and MNs, in parallel with normal, wild-type SOD1 human iPSCs (WTC11), as confirmed by the immuno-detection of OLIG2, Tuj1, and HB9 (Additional file 2: Figure S4a) and by qPCR on OCT4, NANOG, OLIG2, HB9, and CHAT (Additional file 2: Figure S4b). Interestingly, the WTC11 and ALS iPSC cells did not differ in the expression of pluripotency markers or morphology at iPSC stage, or in their MN markers when differentiated (Additional file 2: Figures S2 and S4). Additionally, there was no difference in the derivation efficiency of WTC11-MN and ALS-MN (Additional file 1: Tables S3 and S4).

**Fig. 1:**
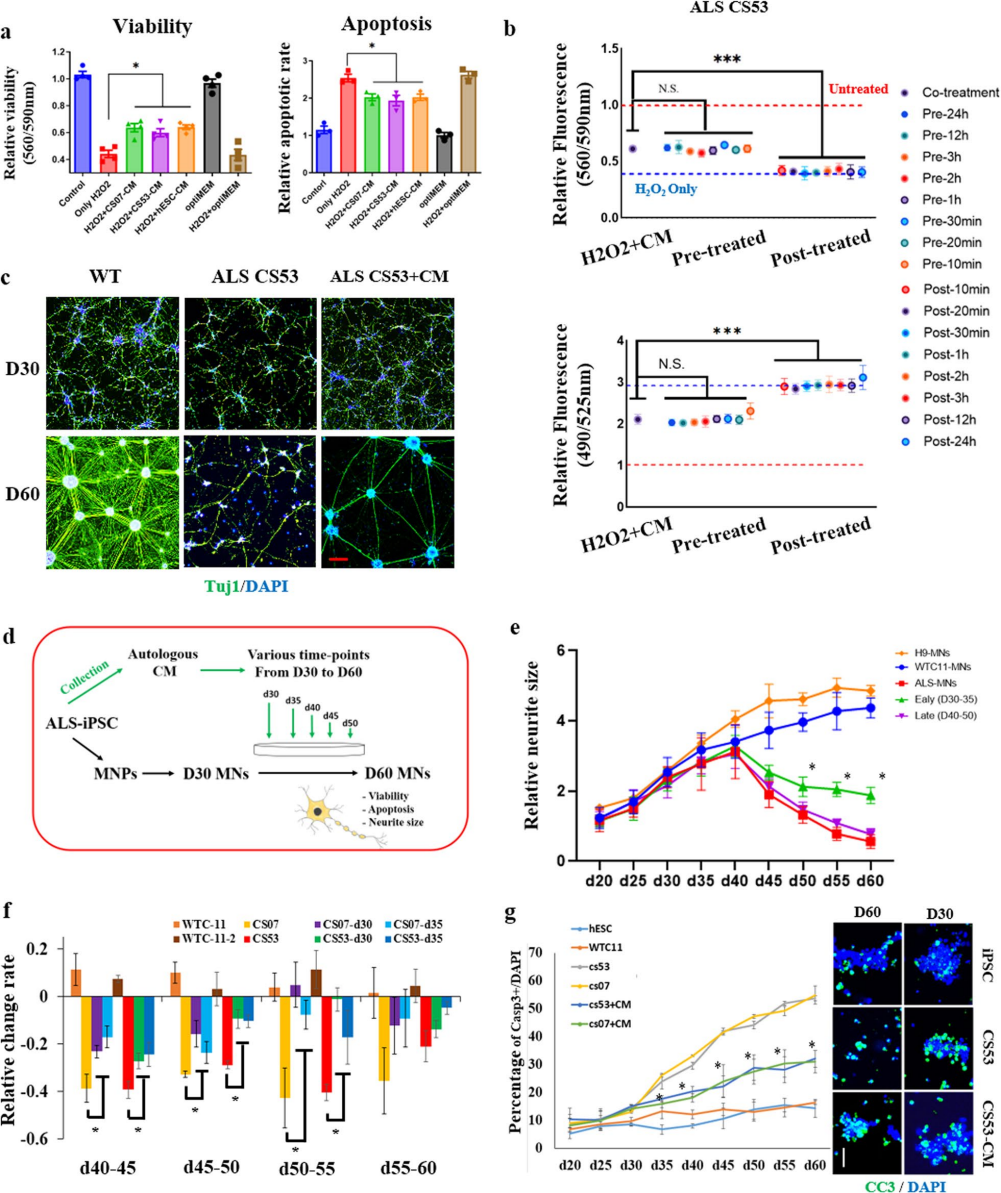
ALS iPSC conditioned medium (CM) is neuroprotective for ALS-MNs. **a** The effect of ALS iPSC-CM on viability and apoptosis in ALS-MNs. The viability of ALS-MNs exposed to H_2_O_2_ was measured by MTT assay. The ratio of apoptotic to live ALS-MNs was assayed by Annexin V fluorescence in a plate reader. *n* = 4, *P < 0.05. **b** The time course of viability (560/590 nm) and apoptosis (490/590 nm) of MNs that were pre-treated or post-treated (H_2_O_2_ first, then ALS iPSC-CM) with ALS iPSC-CM. **c** Representative images of MNs at 30 days and 60 days. WT, WT iPSC differentiated into MNs; ALS CS53, ALS iPSC differentiated into MNs; ALS CS53 + CM, iPSC differentiated into MNs in the presence of ALS iPSC CM. Scale bar, 200 μm. **d** Experimental schematic. The CMs from WT-iPSC, ALS-iPSC, and iPSCs-derived fibroblasts (dF-CM) were used to treat the differentiating MN cultures on days 30–60, after which MN assays were performed. **e** The neurite size was measured during MN differentiation from hESC, iPSC and ALS-iPSCs (± autologous ALS iPSC-CM on days 30, 35, 40, 45, and 50). ALS-MN neurites (CS53 and CS07) were maintained by ALS iPSC-CM when treatment started on day 30 or 35, but not on later days. For the negative control dF-CM, there were no cells that maintained neurites. Mean ± SEM, experiments were repeated with four independently derived MN cell lines from ALS hiPSCs (i.e., *n* = 4). *P < 0.05. **f** The net change of neurite size was examined during differentiation of ALS-MNs treated with autologous ALS iPSC-CM, as indicated. **g** Left, the percent of cleaved caspase-3 (CC3)-positive cells was quantified during this directed differentiation of MNs. Right, representative images of CC3 (green)-positive MNs with DAPI staining of cell nuclei (blue). Mean ± SEM, *n* = 5. *P < 0.05, **P < 0.01, ***P < 0.001. Scale bar, 100 μm

When exposed to H_2_O_2_, MNs had diminished viability and increased apoptosis, and interestingly, all tested PSC CM, hESC-CM, WT iPSC-CM and most importantly the ALS patient-derived iPSC-CM, had neuroprotective effects in this experimental set-up (Fig. 1a). We further explored the timing of this neuroprotective effect of healthy human iPSC-CM and ALS patient iPSC-CM on MNs by either pre-treating the cells with these CMs at variable time points before H_2_O_2_ (Pre-treatment) or alternatively, by treating the cells with H_2_O_2_ first for variable times before adding the CMs (Post-treatment). The viability and apoptotic assays demonstrated that either WT iPSC-CM (Additional file 2: Figure S5) or ALS iPSC-CM (Fig. 1b) was critically needed to be present at least 10 min before H_2_O_2_ was added to protect the MNs from the cytotoxic effects, whereas the addition of iPSC-CMs after H_2_O_2_ (10 min to 24 h) did not confer this neuroprotection.

ALS-MNs are known to show a decrease in the size of their neurites as compared to wild-type MNs [14], and thus we examined this physiologically important parameter in the primary ALS-MN lines (CS53 and CS07) that were treated with their autologous ALS iPSC-CMs. The neurite size increased gradually for 40 days of differentiation of iPSCs into MNs with no difference between the wild-type and ALS cells (Fig. 1c, Additional file 2: Figure S6a and b). However, at 45 days of differentiation and all subsequent time points, the neurite size markedly shrunk in the ALS cohort as compared to the wild-type cells (Fig. 1c, Additional file 2: Figure S6b). The neurite length fluctuation suggested that the time from 40 to 45 days represents a key transition time-point of regulation of neurite outgrowth, where shrinkage becomes prominent in ALS cells.

Considering that pathological changes in the MNs started to manifest at ~ 40 days of their differentiation from the ALS hiPSCs (Additional file 2: Figure S6a, b), the CM was added to the culture medium on day 30, 35, 40 or 45 (Fig. 1d). The assays of neurite size showed that the maintenance of neurites was improved in the groups treated with ALS iPSC-CM starting on either day 30 or day 35 (Fig. 1e and Additional file 2: Figure S6c). Moreover, the rate of neurite size degeneration was significantly reduced by ALS iPSC-CM added on day 30 or 35 in both CS53 and CS07 ALS-MN lines (Fig. 1f). However, when added at later time point, e.g., on day 40, ALS iPSC-CM was not able to rescue the neurite size of ALS-MNs (Fig. 1e and Additional file 2: Figure S6c).

We also analyzed the relative numbers of apoptotic cells in CS53 and CS07 ALS-MNs. There was a slight upward trend in the number of CC3-positive cells between the wild-type and ALS-MN lines up to day 30, but from day 35 onward the number of CC3-positive cells started to dramatically increase in the ALS-MN cultures as compared to the healthy MN cultures (Fig. 1g and Additional file 2: Figure S6d). Notably, the rate of ALS-MN apoptosis was significantly attenuated by the autologous ALS iPSC-CM, for both ALS-MN cell lines (Fig. 1g and Additional file 2: Figure S6e).

These data reveal that ALS patient iPSC-CM has multi-functional neuro-protective effects on their ALS-MNs, improving cell viability and the maintenance of neurites.

### Neuroprotection, improved neuro-muscular function, and delayed morbidity of ALS mice bearing the human disease SOD1^G93A^

To determine whether the CMs from ALS patient-iPSCs could have the same neuro-protective effects *in vivo*, we used transgenic mice engineered to carry the mutant human SOD1 (SOD1^G93A^) gene, which display progressive degeneration of MNs and the phenotypes of ALS and are used extensively to study human ALS [29, 34, 35], to assess the effects of ALS iPSC secretome *in vivo*. The first symptoms occurred in these mice at 80–90 days of age and morbidity at 128 ± 9 days [36]. So we studied from the asymptomatic stage (68 days) through the entire progression of the disease to the morbidity endpoint.

Twelve ALS mice were studied for the effects of ALS iPSC-CM (CS07 and CS53) and 6 ALS mice were studied in parallel, using the negative control, dF-CM. Age-matched males and females were identically treated with either ALS iPSC-CM or dF-CM. Throughout the study, there was no significant difference in body weight between ALS iPSC-CM-treated and the dF-CM-treated mice (Additional file 2: Figure S7).

**Fig. 2:**
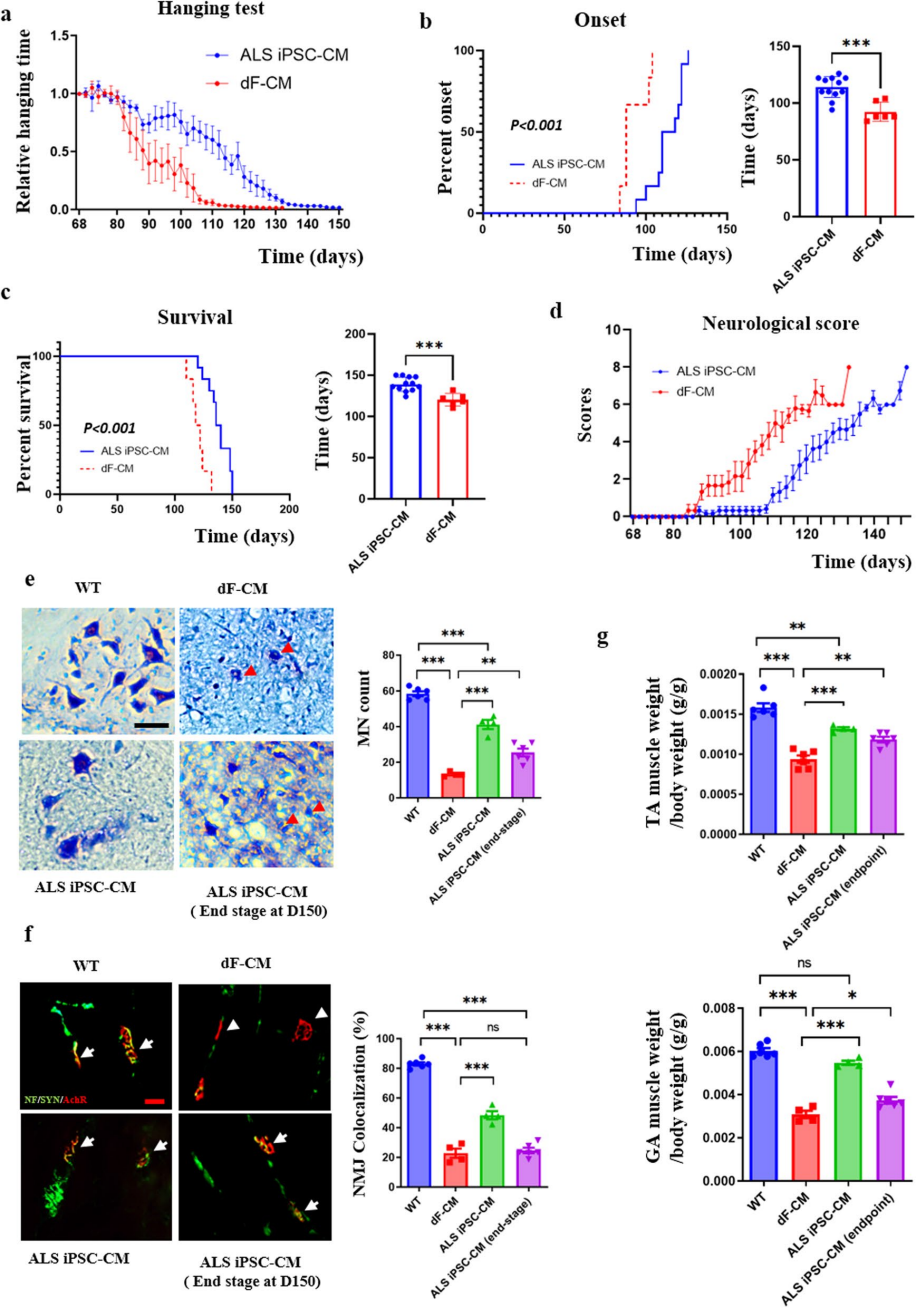
*In vivo* neuroprotection of ALS iPSC secretome in the *SOD1*^G93A^ mouse ALS model. **a** Hanging test of the transgenic mice (ALS iPSC-CM-treated mice *n* = 12 and dF-CM-treated mice *n* = 6). The decline in performance was significantly delayed in the mice treated with ALS iPSCs-CM, as compared to the dF-CM (114.8 ± 4.29 days *vs* 91.0 ± 4.31 days, *P* < 0.01). Values are mean ± SEM. **b** Kaplan–Meier curves of the disease onset showing that onset was delayed in the ALS iPSC-CM, as compared to the dF-CM group. Onset: (116.8 ± 3.64 days *vs* 92.3 ± 3.44 days, *P* < 0.001). **c** Kaplan–Meier curves of animal survival showing that the ALS iPSC-CM increased lifespan, as compared to dF-CM (141.5 ± 3.26 days *vs* 120.3 ± 3.07 days, *P* < 0.001). **d** Neurological scores were found to be improved by the ALS iPSC-CM, as compared to dF-CM. **e** Histological analysis (WT for SOD1 C57.B6 mice, *n* = 6, and ALS mice, *n* = 8) at 120 days of age, and at the end stage (morbidity time point). Representative images for Cresyl violet staining (and their quantification, bar graph) in the ventral lumbar spinal cord at 120 days showing that ALS iPSC-CM improved the number and the morphology of motor neurons of ALS mice, as compared to the dF-CM. Arrowheads, shrunken MNs. Scale bar, 200 μm. **f** Representative images of immunostaining of NMJ in gastrocnemius muscle at 120 days and quantification showing that the percent of colocalized NMJ decreased in the dF-CM group, as compared to the WT mice, whereas this percent was improved in the ALS iPSC-CM group. Arrows, colocalized endplates; arrowheads, mis-localized endplates. NF, Neurofilament, SYN, Synaptophysin, AchR, Acetylcholine receptors. Scale bar, 100 μm. **g** Mass of tibialis anterior (TA) and gastrocnemius (GA) muscles was also increased in the ALS mice treated with ALS iPSC-CM as compared to dF-CM

The control dF-CM-treated mice showed rapid deterioration of neuro-muscular function, as measured through neurological scoring [29] and four-limb hanging tests [37]. Remarkably, the ALS iPSC-CM rescued the agility, coordination, and overall muscle function of the *SOD1* mutant mice, delayed the onset of pathological symptoms (Fig. 2a, b), delayed morbidity (Fig. 2c), and improved the neurological scores of the ALS model animals (Fig. 2d). Consistent with the neuro-muscular improvements, survival was significantly prolonged in the ALS iPSC-CM group as compared to the dF-CM group (141.5 ± 3.26 days *vs* 120.3 ± 3.07 days, *P* < 0.001) (Fig. 2c).

To investigate the effect of ALS iPSC-CM on the lumbar spinal cord and neuromuscular junction, these structures were analyzed postmortem in 120-day-old mice. Compared to the C57.B6 wild-type animals, the SOD1^G93A^ mice with dF-CM treatment showed decreased total number of cresyl violet-stained MNs in the ventral spinal cord (58.5 ± 3.33 *vs* 13.5 ± 1.25, *P* < 0.001), indicating progression of the disease (Fig. 2e), and many cresyl violet-stained MNs showed shrunken morphology with no neurites (Fig. 2e). However, the number of MNs was significantly increased in the SOD1^G93A^ mice treated with ALS iPSC-CM, as compared to the dF-CM group (41.25 ± 5.12 *vs* 13.5 ± 1.25, *P* < 0.001), which was also observed at the end-stage (at 150 days; 25.50 ± 5.32 *vs* 13.5 ± 1.25, *P* < 0.05; Fig. 2e). These results demonstrate that the treatment of ALS model mice with ALS iPSC-CM prevents deterioration of spinal MNs.

NMJ defects contribute to the decline in muscle mass and health [37], and NMJ alterations are a feature of neurological diseases [38–40]. Thus, we investigated NMJ integrity and muscle innervation, using the specific markers acetylcholine receptor, neurofilament and synaptophysin. Our results showed that the number of NMJs was significantly reduced in the dF-CM group, as compared to the C57.B6 group at day 120 (Fig. 2f), while the ALS iPSC-CM group had significantly more intact NMJs than the dF-CM group (48.46 ± 5.43 *vs* 22.87 ± 6.12, *P* < 0.001). As expected, at the end-stage, there was no difference between the dF-CM and the ALS iPSC-CM groups (Fig. 2f). These findings suggest that the treatment of SOD1^G93A^ mice with ALS iPSC-CM preserves muscle innervation for a longer time, which is consistent with the improved performance in four-limb handing test and better neurological scores.

Improved NMJ integrity suggests a reduction in muscle atrophy, so we determined the weights of gastrocnemius anterior and tibialis anterior of the mice. The mass of gastrocnemius and tibialis anterior was normalized to the body weight. As expected, there was a significant decrease in the average weights of gastrocnemius and tibialis anterior in the dF-CM group, compared to the wild-type C57.B6 group. However, these weights were significantly increased in the ALS iPSC-CM group, as compared to the dF-CM group (Fig. 2g).

In summary, these data demonstrate that the ALS iPSC-CM significantly delays the onset of paralysis and functional decline in the SOD1^G93A^ mice, extends their lifespan and protects MNs, NMJs and muscle mass. Such profound multiparametric positive effects  in ALS, averting and delaying all key manifestations of this deadly disease, have not been reported previously with other tested methods.

### Mechanisms, relation to pluripotency, sub-cellular details and improvement over CsA

**Fig. 3:**
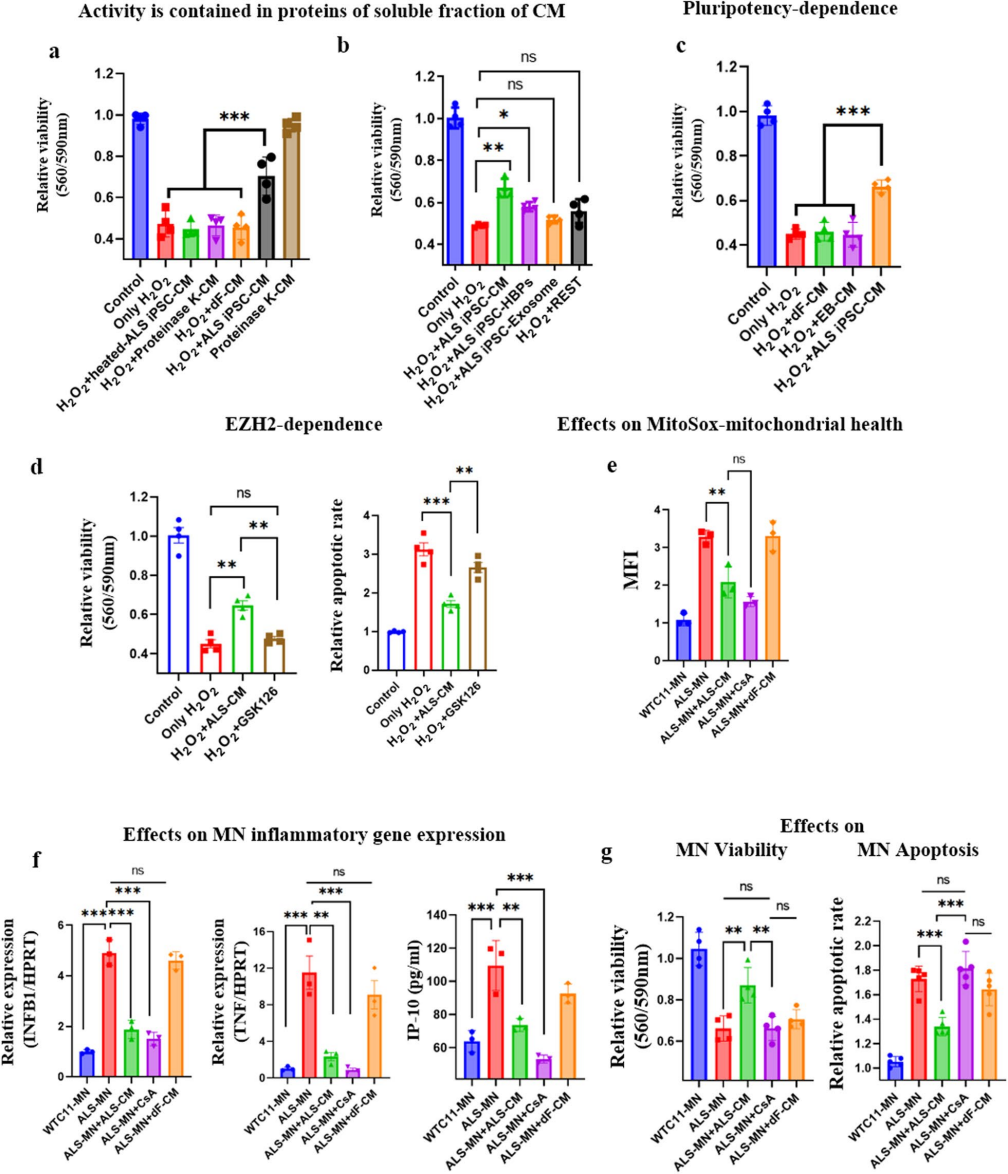
Mechanisms of neuroprotection by ALS iPSC-CM. **a** MTT assay for viability of MNs cultured with proteinase K-treated or heat-inactivated ALS iPSC-CM. ***P < 0.001, *n* = 4 for each condition. **b** MTT assay for viability of MNs that were cultured with 200 μg/ml H_2_O_2_ alone or co-treated with complete unfractionated ALS iPSC-CM, heparin-binding protein fraction, exosome fraction or HPB flow-through and exosome-free CM. *P < 0.05, **P < 0.01, *n* = 4 for each condition. **c** MTT assay for viability of MNs that were cultured with secretome of ALS iPSC-derived embryoid bodies (EBs). The viability was significantly decreased in the EB-CM group compared to that in the ALS iPSC-CM group (0.66 ± 0.02 *vs* 0.44 ± 0.05, *P* < 0.001). ***P < 0.001, *n* = 4 for each condition. **d** The effect of epigenetic modifiers on the neuroprotective activity of the iPSC secretome: ALS iPSCs were treated with the indicated inhibitors of pluripotency that influence epigenetics, and the activity of their CM was subsequently tested with H_2_O_2_-exposed ALS-derived MNPs, in quadruplicate MTT and Annexin V assays. EZH2 inhibitor GSK126 diminished the neuroprotective activity of the iPSC-CM with high statistical significance (viability: 0.64 ± 0.04 *vs* 0.47 ± 0.02, *P* < 0.01; apoptosis: 1.71 ± 0.17 *vs* 2.65 ± 0.23, *P* < 0.01), *P < 0.05, **P < 0.01, ***P < 0.001, *n* = 4 for each condition. **e** Levels of mitochondrial ROS. All MNs were stained with mitoSOX red and then the mean fluorescence intensity (MFI) was measured by a Guava Easycyte Flowcytometer. Autologous ALS iPSC-CM normalized mitochondrial activity of ALS-MNs statistically same as the known modifier, Cyclosporin A (CsA). Graphs show Mean ± SEM, *n* = 4. **P < 0.01. **f** The effects on inflammation were assayed by qRT-PCR. The indicated markers of inflammation were diminished by ALS iPSC-CM similarly to CsA. *n* = 3, ***P < 0.001, *ns* not statistically significant. *n* = 4, **P < 0.01, ***P < 0.001, *ns* not statistically significant. **g** The effect of CsA on MN viability and apoptosis. The viability of ALS-MNs as compared to healthy WTC-11 MNs was measured by MTT assay. The ratios of apoptotic to live MNs were assayed by Annexin V fluorescence in a plate reader. ALS-MNs were prone to cell death, as compared to WTC-11 MNs_;_ ALS iPSC-CM, but not dF-CM or CsA, protected ALS-MNs from the ROS (mutant SOD1)-induced cell death

To understand the mechanisms by which the iPSC secretome (CS07) is neuroprotective in ALS, we performed biochemical characterizations. First, we investigated whether the effect was produced by proteins. Heat inactivation and proteinase K treatments abrogated the positive effects of the iPSC secretome, demonstrating that the neuroprotective activity is contained in the protein fraction (Fig. 3a).

Several reports have shown that the exosomes from stem cell-CM have a protective effect on cellular damage/stress [26, 41]. Our previous studies documented that heparin-binding proteins from hESC-CM promote proliferation of muscle and neural cells [19, 20]. Accordingly, we examined whether the neuroprotective activity is contained in the heparin-bound (HB)* versus* unbound soluble fractions and exosome fraction. First, we determined the maximum amount of HB fraction tolerated by cells, and the HB fraction became cytotoxic at 300 μg/ml (Additional file 2: Figure S8a). Exosomes, purified from hESC-CM by ultracentrifugation [26], negatively affected cell viability at 200 μg/ml (Additional file 2: Figure S8b). Based on these data, 200 μg/ml heparin-binding proteins and 100 μg/ml exosome preparations were used to treat MNs. There was an increase in the viability of human MNs that were co-treated with H_2_O_2_ and HB fraction, as compared to H_2_O_2_ alone (0.58 ± 0.02 *vs* 0.49 ± 0.01, *P* < 0.05); however, the efficiency of that neuroprotection was less than that displayed by the unfractionated ALS iPSC-CM (0.66 ± 0.04 *vs* 0.58 ± 0.02, *P* < 0.05) (Fig. 3b). There was no improvement in viability or decrease in apoptosis of MNs that were co-treated with H_2_O_2_ and the exosome fraction (Fig. 3b). The soluble fraction that did not contain exosomes or HB proteins, had a slight, not statistically significant neuroprotection (Fig. 3b). These data suggest that the neuro-protective activity is contained in multiple fractions of PSC-CM, and mostly in the soluble fractions (heparin-bound and-unbound).

Based on the absence of neuroprotective activity in dF-CM, we decided to confirm and mechanistically extrapolate the relationship between the protective effects and pluripotency. As shown in Fig. 3c, differentiation of PSCs into embryoid bodies negated the neuroprotective activity, expanding the data on PSC-derived fibroblasts and confirming that a state of pluripotency is required for the neuroprotective activity. To follow on the requirement for pluripotency, we employed a screen with inhibitors of pluripotency that modify the epigenetic states of iPSCs. We treated separate cultures of ALS iPSCs with the inhibitors: Nanomycin A, LSD1 inhibitor, 5 Azacytidine, or GSK126 (Fig. 3d and Additional file 2: Figure S9). After 3 days in culture with each of these small molecules, the expression of pluripotency markers decreased (Additional file 2: Figure S9a). However, pluripotent colony-like morphologies were maintained, and *OCT4* and *NANOG* expression was higher in each single inhibitor culture, as compared to the embryoid bodies differentiated from iPSCs (Additional file 2: Figure S9a and b). To determine the effects of epigenetic modifiers on the neuroprotective properties, we studied the viability and apoptosis of MNPs exposed to H_2_O_2_ that were pre-treated with CM from ALS iPSC exposed to the epigenetic inhibitors. Interestingly, treatment of ALS iPSCs with EZH2 inhibitor, GSK126, significantly reduced the neuroprotective activity of the ALS iPSC-CM (Fig. 3d), while other inhibitors showed  no difference or non-statistical trend (Additional file 2: Figure S9c). This suggests the possibility that an EZH2-dependent pathway regulated by GSK126 is important for the neuroprotective activity of the ALS iPSC secretome.

To understand the sub-cellular effects of the ALS iPCS secretome on MN, we assayed mitochondrial health, which is known to diminish in ALS-MNs, and generally in neurons of patients with neurological diseases [42, 43]. Specifically, we performed the mitoSox red mitochondrial ROS analysis on ALS and healthy MNs, and on the ALS-MNs that were cultured with the ALS iPSC-CM as compared to the dF-CM (Fig. 3e). As expected, ALS-MNs had higher mean fluorescence intensity (MFI) of mitoSox red as compared to the healthy MNs, and the positive control, treatment with immunosuppressive CsA that blocks mitochondrial pores, reduced the MFI (Fig. 3e). Notably, autologous ALS iPSC-CM significantly reduced the MFI of mitoSox red, as compared to the untreated or dF-CM-treated MN, to a degree that was statistically indistinguishable from CsA (Fig. 3e).

We also explored the relationship of ALS iPSC-CM to neuroinflammation, the shared phenotype of many neurological disorders, including ALS [44–46]. ALS iPSC-CM significantly diminished the expression of pro-inflammatory INFB1, TNF-α, and IP-10 in ALS-MNs, and these effects were similar in the CsA-treated ALS-MNs (Fig. 3f).

Since CsA and ALS iPSC-CM both improved mitochondria and reduced expression of INFB1, TNF, and IP-10, we compared them side-by-side for neuroprotection in diminishing apoptosis and improving the viability of human ALS-MNs. Interestingly, only ALS iPSC-CM, but not CsA, reduced apoptosis and increased viability of the SOD1 mutant ALS-MNs that were derived from patients with this disease (Fig. 3g).

These results establish that the ALS iPSC-CM activity is mediated by multiple soluble secreted proteins (not exosomes), which likely act together since fractionation results in diminished activity of every fraction, as compared to the unfractionated CM. The ALS iPSC-CM activity is dependent on the state of pluripotency and in an EZH2-dependent process; it improves mitochondrial health and reduces neuro-inflammatory gene expression. These data also show that while CsA blocks mitochondrial pores and both CsA and ALS iPSC-CM stabilize mitochondria, there are additional effects of ALS iPSC-CM on averting the death of ALS-MNs.

### Identification of a neuro-protective protein interactome

**Fig. 4:**
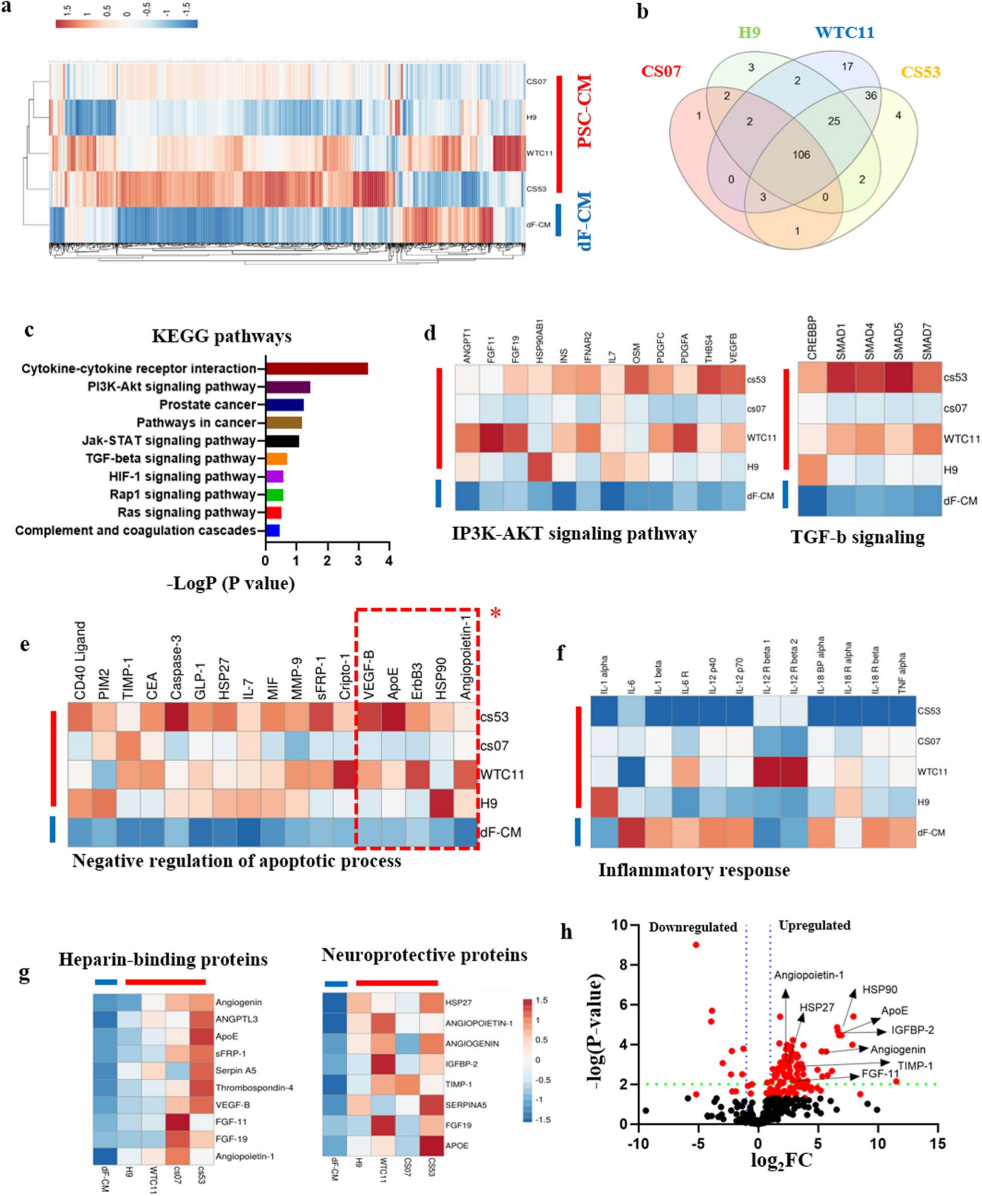
Proteomic profiling analysis. **a** Heat mapping of WTC11, H9, CS07, CS53, and dF-CM groups. The dF-CM is farthest from the other groups. **b** The Venn diagram of the proteins that were present at different levels in studied conditioned media. **c** The KEGG pathway list according to biological functions. **d** Heat mapping of representative KEGG pathways, iP3K-AKT signaling pathway (*P* = 5.00E−04) and TGF-β signaling pathway (*P* = 9.30E−03). **e** Heat mapping of negative regulators of apoptotic process (*P* = 3.00E−06) and inflammatory response (*P* = 2.60E−06). *Negative regulation of neuronal apoptosis (*P* = 8.00E−03). **f** Heat mapping of inflammation-related proteins. The levels of these proteins were lower in PSC-CM than in dF-CM. **g** Heat mapping of Heparin-bound proteins and neuroprotective proteins. **h** Volcano plot of the PSC-CM and dF-CM. The red dots represent differently expressed proteins (*P* < 0.05; FC > 1.75), while the grey dots represent proteins with *P* > 0.05

To identify candidate neuroprotective proteins, we performed comparative proteomic antibody capture arrays that detect 1000 factors, comparing hESC-CM, WT iPSC-CM and ALS iPCS-CM (CS07 and CS53) that all had neuroprotective activity, with each other and with the dF-CM that lacked neuroprotective activity (Fig. 4a). This screen identified 106 candidates (Venn diagram, Fig. 4b) that were up-regulated in all PSC-CM groups, as compared to the dF-CM (> twofold change with *P* < 0.05). The violin plot showed that the proteome patterns of hESC-CM, WT iPSC-CM and ALS iPSC-CM were significantly different from those of dF-CM (Additional file 2: Figure S10a). The list of KEGG pathways was constructed through grouping the proteins by their key functions (Fig. 4c). PSC-CMs and dF-CMs significantly differed in the levels of regulatory proteins that participate in canonical morphogenic signaling pathways, including PI3K-Akt, Jak-STAT, BMP/TGF-β, and Ras, all of which were shown to be important for development, viability and/or maintenance of neurons (Fig. 4d) [47].

Additional protein characterizations according to the biological process, molecular function, and cellular component by DAVID (Additional file 2: Figure S10b) showed that consistent with the functional data, the biological process group had 170 GO terms, including positive regulation of cell proliferation, signal transduction, positive regulation of peptidyl-tyrosine phosphorylation, negative regulation of apoptosis, control of wound healing, and beta-amyloid clearance; the latter highly consistent with our previously reported [20] neuroprotection from ectopic beta-amyloid (Fig. 4e and Additional file 2: Figure S10b).

One of the most important causes of MN degradation is the inflammatory response [44–46]. The proteomic screening showed that several proteins and cytokines known to induce inflammation, including IL-1, IL-6, IL-12, and TNF-α, were reduced in PSC-CMs compared to the dF-CM (Fig. 4f).

Interestingly, in concert with the above fractionation experiments and our previous findings on heparin-binding activity of pro-regenerative hESC-CM [19, 20], we identified several heparin-binding proteins that were elevated in the PSC-CM cohorts as compared to the dF-CM, including angiogenin (ANG), Angiopoietin-1 (ANG-1), angiopoietin like 3, apolipoprotein E (ApoE), secreted frizzled related protein 1, serpin family A member 5, thrombospondin 4, vascular endothelial growth factor B, fibroblast growth factor 11, and fibroblast growth factor 19 (Fig. 4g). Most of these heparin-affinity factors promote cell proliferation and attenuate cell death [48–51].

Among proteins that do not bind to heparin, but were identified in the neuroprotective PSC secretome (Fig. 4g), TIMP1 has shown protective effects against not only traumatic and ischemic brain injury but also HIV-1-induced neuronal apoptosis [51, 52]. IGFBP-2 is involved in regulation of cell proliferation and has an anti-apoptotic effect* via* modulation of CC3 [53, 54]. IGFBP-2 may have a role in neuroprotection from the hypoxic–ischemic injury and protects neurons from beta-amyloid-induced toxicity [55, 56]. HSP27 has an intrinsic responsibility in neuroprotection by increasing cell viability and decreasing apoptotic signaling [57–59]. Comparing PSC-CM and dF-CM through volcano plots, the identity of proteins which may play important roles in various aspects of neuroprotection was determined (Fig. 4h).

The comprehensive comparative proteomics suggests that activities of multiple ALS iPSC-secreted proteins have the capacity, in concert, to avert the degeneration and death of ALS-MNs: some proteins inhibit apoptosis by mechanisms independent from each other, while others reduce protein aggregation, reduce ROS damage, improve post-ROS repair, and/or support neuronal cell fate.

## Discussion

This work demonstrates that proteins secreted by PSCs are neuroprotective and importantly, that the ALS patient-derived iPSCs can be used to combat their disease. Induced oxidative stress (such as by H_2_O_2_) is known to induce the death of many types of neuronal and non-neuronal cells. In sporadic and familial ALS, mutations in genes of different functions, promote oxidative stress, which is thought to contribute to neurodegeneration [4, 5, 60, 61]. Thus, we first established that hESC-CM was able to protect fibroblasts and MNs from H_2_O_2_-induced apoptosis. This neuroprotective activity was then confirmed with ALS patient hiPSC-CM. H_2_O_2_ is an extreme neurotoxin, hence it is important that in various screens and all independent experiments, PSC-CMs consistently enhanced the viability of MNs and diminished their apoptosis by ~ 20% in the presence of H_2_O_2_. Next, we established that proteins secreted by the WT and ALS hiPSCs promote cell viability, formation and stability of neurites, and mitochondrial health of *SOD1* mutant human ALS-MNs. *In vivo*, human ALS iPSC-CM delayed the onset of symptom and extended the lifespan of *SOD1* mutant ALS mice, through improved maintenance of MNs and NMJs. ALS iPSC-CM could slow down, but not overcome the progression of ALS, which might be improved when the defined proteins are determined and tested in future work.

The autologous patient’s iPSC secretome avoids the problems of cell transplantation: incomplete differentiation, danger of transplanting cancer-causing cells and difficulty of iPSC maturation [62–64]. Moreover, the autologous approach using the patient’s own hiPSCs will minimize the variability of heterologous hiPSCs and the immune response to heterologous proteins. Reprogramming of disease-specific differentiated cells into iPSCs often results in normal pluripotency, even when there are disease-associated mutations, and in our work, ALS-iPSCs were typical pluripotent stem cells, based on their markers and the efficiency of MN differentiation [65–67].

Mechanistically, our results suggest that the activity is protease-sensitive and that a combination of proteins from different biochemical fractions are needed for the neuroprotection (Fig. 3a). Based on our comparative proteomics, the candidate proteins that were enriched in the PSC-CM as compared to the inactive dF-CM, are either secreted or can be secreted when cells are damaged, and/or* via* exosomes or microvesicles [68, 69] (Additional file 1: Table S5). Because the neuroprotective activity of the hiPSC secretome is diminished when either exosomal or soluble fractions are removed, both fractions likely have proteins that confer neuroprotection through their interactome.

**Fig. 5:**
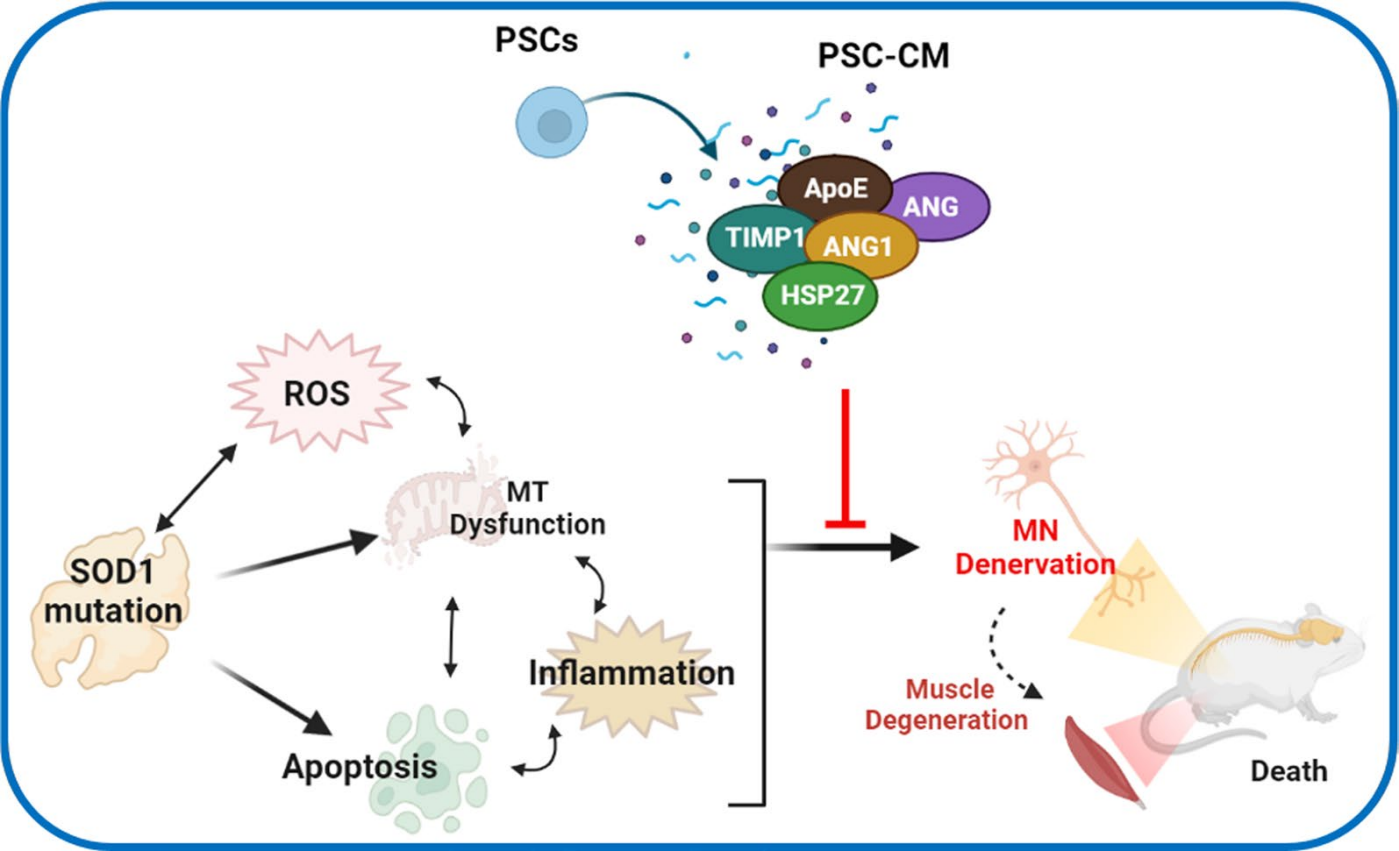
Mechanisms of multifunctional neuroprotection through PSC-CM. Oxidative stress alters SOD1 activity, and dysfunctional or mutant SOD1 causes further oxidative damage; this induces apoptosis, destabilizes mitochondria and promotes hyperinflammation. Distinct PSC-CM proteins through different mechanisms have the capacity to act together to attenuate ROS, improve proteostasis, inhibit Cas3 activation and apoptosis, and promote neuronal repair. The key candidates in PSC-CM as neuroprotective factors are TIMP1, ApoE, ANG, ANG1, and HSP27, whose neuroprotective effects have been confirmed in literature. Ultimately, PSC-CM delays or inhibits death and denervation of MNs, promoting muscle innervation and preventing degeneration. Figure created using BioRender (https://biorender.com/)

Uncovering the specific interactions of specific proteins that are necessary and sufficient is clearly a subject of future work. At the same time, several neuroprotective candidates seem to be particularly promising, and Fig. 5 summarizes their literature-predicted activities. TIMP1, ANG, ANG1, HSP27, and APOE were enriched in PSC-CM as compared to the negative control dF-CM, and each of these proteins has known neuroprotective effects [50–52, 70–77].

TIMP1 protects neurons against not only traumatic and ischemic brain injury but also HIV-1-induced neuronal apoptosis [51, 52]. Angiogenic ANG [78, 79] is necessary for success in maturation of neurons, including MNs in the spinal cord and brain [80, 81], protects MNs from hypoxia, and contributes to enhanced viability of MNs and longer lifespan of ALS mice [72, 73]. ANG-1 is another angiogenic factor in the secretomes of stem cells [79, 82, 83] that has anti-inflammatory [84] and neuroprotective anti-apoptotic effects [74–76]. HSP27 is an antioxidant and inhibitor of procaspase-3 activation [85–87], and these activities are being explored in Alzheimer’s disease [59, 88] and ALS models [70, 77]. ApoE reduces oxidative stress and contributes to beta-amyloid disposal [50, 71]. Moreover, all these proteins interact at the levels of apoptotic, TGF-β, AKT, and ERK signaling pathways [72–76, 87], suggesting a possible functional synergy.

Several clinical trials have suggested positive effects of mesenchymal and adipose stem cell secretomes [21, 89–92], but interestingly, here we showed that pluripotency was required for the positive effects and that differentiation of PSCs into fibroblasts or even into embryoid bodies abrogated the neuroprotection. Epigenetic modification is one of the major regulators of pluripotency [93]. Our study demonstrated an EZH2-mediated mechanism of the neuroprotective effect. EZH2 is a key part of the Polycomb repressive complex, histone methyltransferase [94]. The expression of EZH2 is not only required for pluripotency [95, 96], but influences differentiation into various cell fates, including neurons [97–99]. And EZH2 depletion has been shown to induce cell cycle arrest, decrease cell proliferation and induce apoptosis [100, 101]. Consistently, we found that GSK126 treatment of iPSCs reduced the neuroprotective anti-apoptotic activity of their secretome. Interestingly, some of the proteins identified in our comparative proteomics crosstalk with EZH2. For example, HSP90 is involved in EZH2 stability, level and function [102, 103]; ANG-1 and HSP27 are regulated by EZH2 and their expression is decreased upon EZH2 inhibition [104, 105]. The conclusion that the state of pluripotency is needed for the neuroprotective effects of PSC secretome fits well with the notion that pluripotent stem cells maintain low levels of oxidative stress* via* oxidation–reduction (redox) signaling, as compared to their differentiated progeny [106–109].

At the cellular level, ALS iPSC-CM improved the mitochondrial stability of MNs and reduced the expression of inflammatory genes. Recently suggested as an ALS treatment, CsA [46] had similar effects on mitochondrial health and MN-expressed inflammatory genes, but in contrast to ALS iPSC-CM, CsA failed to prevent the death of ALS *SOD1* mutant MNs (Fig. 3e–g). CsA is also an immunosuppressant. The fact that ALS iPSC-CM stabilizes mitochondria and the hyperinflammatory response in ALS-MN without immunosuppression potentially makes it a better therapeutic.

In addition, the lack of neuroprotective effect of late application of ALS iPSC-CM suggests that an early diagnosis and immediate treatment are needed for the optimal efficacy of PSC-CM and the autologous ALS iPSC-CM in attenuating the pathologies, which is the case with therapies for most neurological diseases.

## Conclusion

Neuronal cell death is causal in many neurodegenerative diseases, including age-related loss of memory and dementias (such as Alzheimer’s disease), Parkinson’s disease, strokes, as well as diseases that afflict broad ages, i.e., traumatic brain injury, spinal cord injury, ALS, and spinal muscle atrophy. These diseases are characterized by neuroinflammation and oxidative cell damage, many involve perturbed proteostasis and all are devastating and without a cure. Our work describes a feasible meaningful disease-minimizing treatment for ALS and suggests a clinical capacity for treating a broad class of diseases of neurodegeneration, and excessive cell apoptosis [14, 110–113].

## Supplementary Information

**Additional file 1**. **Table S1**. Primer sequences for real-time polymerase chain reaction. **Table S2.** The list of amyotrophic lateral sclerosis cell lines. **Table S3**. The efficiency of motor neuron differentiation from normal pluripotent stem cells. **Table S4.** The efficiency of motor neuron differentiation from ALS patient hiPSCs. **Table S5. **The 106 PSC-CM protein candidates and their secretory capacity.

**Additional file 2**. **Figure S1.** Human embryonic stem cell-derived conditioned medium (hESC-CM) protects human fibroblast from H_2_O_2_ cytotoxicity. **Figure S2.** Human embryonic stem cell-derived conditioned medium (hESC-CM) protects motor neurons (MNs) from H_2_O_2_ cytotoxicity. **Figure S3.** Antioxidant activity is not different between human embryonic stem cell-derived conditioned medium (hESC-CM) and differentiated fibroblast-derived conditioned medium (dF-CM). **Figure S4.** Differentiation of ALS iPSCs into motor neurons (ALS-MNs). **Figure S5.** Profiling the time-point of the neuroprotective effect from iPSC-CM. **Figure S6.** The comparative effects of autologous ALS-derived hiPSC-conditioned media (ALS-CM) on neurites and apoptosis of ALS-motor neurons (ALS-MNs) in ALS patient-derived cell lines. **Figure S7. **The effect of CMs on the weight of SOD1^G93A^ transgenic mice. **Figure S8.** The optimization of concentrations of heparin-binding protein (HBP) and exosome. **Figure S9.** The effect of epigenetic modifiers on the conditioned medium (CM). **Figure S10.** Differentially present proteins in each of the listed categories.

## Data Availability

All data required in the paper, including the primary raw data are provided in the original manuscript and supplementary material file.

## Abbreviations

ALS: Amyotrophic lateral sclerosis
PSC: Pluripotent stem cell
MN: Motor neuron
SOD1: Superoxide dismutase 1
ESC: Embryonic stem cell
iPSC: Induced pluripotent stem cell
CM: Conditioned medium
CsA: Cyclosporin A
MNP: Motor neuron precursor
NBM: Neurobasal medium
PCR: Polymerase chain reaction
NMJ: Neuromuscular junction
dF-CM: Conditioned medium from differentiated fibroblast cells
CC3: Cleaved caspase3
MFI: Mean fluorescence intensity
