# Supplementary material for: Autologous treatment for ALS with implication for broad neuroprotection
Source: Transl Neurodegener. 2022 Mar 11;11:16. doi: 10.1186/s40035-022-00290-5 (PMC8915496; doi:10.1186/s40035-022-00290-5)
Supplement: Supplementary file 1 — Additional file 1. Table S1. Primer sequences for real-time polymerase chain reaction. Table S2. The list of amyotrophic lateral sclerosis cell lines. Table S3. The efficiency of motor neuron differentiation from normal pluripotent stem cells. Table S4. The efficiency of motor neuron differentiation from ALS patient hiPSCs. Table S5. The 106 PSC-CM protein candidates and their secretory capacity. [file 40035_2022_290_MOESM1_ESM.docx]

**Table S1. Primer sequences for real-time polymerase chain reaction**

| **Group** | **Gene name** | **Sequence** |
| --- | --- | --- |
| **Pluripotency** | hOCT4_F | GGGCTCTCCCATGCATTCAAAC |
|  | hOCT4_R | CACCTTCCCTCCAACCAGTTGC |
|  | hNANOG_F | TGGGATTTACAGGCGTGAGCCAC |
|  | hNANOG_R | AAGCAAAGCCTCCCAATCCCAAAC |
| **Motor neuron precursor** | OLIG2_F | GTT CTC CCC TGA GGC TTT TC |
|  | OLIG2_R | AGA AAA AGG TCA TCG GGC TC |
| **Motor neuron** | HB9_F | GTC CAC CGC GGG CAT GAT CC |
|  | HB9_R | TCT TCA CCT GGG TCT CGG TGA GC |
|  | CHAT_F | GGA GGC GTG GAG CTC AGC GAC ACC |
|  | CHAT_R | CGG GGA GCT CGC TGA CGG AGT CTG |
|  | ACTB_F | TGA AGT GTG ACG TGG ACA TC |
|  | ACTB_R | GGA GGA GCA ATG ATC TTG AT |
| **Inflammation** | IFNB1_F | TGTCGCCTACTACCTGTTGTGC |
|  | IFNB1_R | AACTGCAACCTTTCGAAGCC |
|  | TNF_F | TCTCTCAGCTCCACGCCATT |
|  | TNF_R | CCCAGGCAGTCAGATCATCTTC |
| **Housekeeping gene** | HPRT_F | TCAGGCAGTATATCCAAAGATGGT |
|  | HPRT_R | AGTCTGGCTTATATCCAACACTTCG |

The sequences of the primers for the qRT PCR experiments are provided.

**Table S2. The list of amyotrophic lateral sclerosis cell lines**

| **ALS-iPSC Line Name** | **Parent Cell Type** | **Mutation** | **Sex** | **Age at Sample Collection** |
| --- | --- | --- | --- | --- |
| CS07iALS-SOD1A4Vnxx | Fibroblast | SOD1 – A4V (A5V) | Female | 40 |
| CS53iALS-SOD1A4VNTnxx | Peripheral blood mononuclear cell | SOD1 – A4V (A5V) | Male | 35 |

The names, cell origin and mutations of the cell lines that were used in this study.

**Table S3. The efficiency of motor neuron differentiation from normal pluripotent stem cells.**

| **H9** | | | **WTC11** | | |
| --- | --- | --- | --- | --- | --- |
| **# of DAPI** | **# of** HB9 **positive** | **%** | **# of DAPI** | **# of** HB9 **positive** | **%** |
| 11270 | 10612 | 94.16 | 15642 | 14543 | 92.97 |
| 11130 | 10290 | 92.45 | 11818 | 10573 | 89.47 |
| 9654 | 8982 | 93.04 | 6492 | 6039 | 93.02 |
|  | | **93.21±0.86** |  | | **91.82±2.03** |

Comparative efficiencies of the directed differentiation of MNs from the hESC and hiPSC lines.

**Table S4. The efficiency of motor neuron differentiation from ALS patient hiPSCs**

| **CS07** | | | **CS53** | | |
| --- | --- | --- | --- | --- | --- |
| **# of DAPI** | **# of double positive** | **%** | **# of DAPI** | **# of double positive** | **%** |
| 18909 | 17273 | 91.35 | 20094 | 18149 | 90.32 |
| 6316 | 5776 | 91.45 | 11520 | 10430 | 90.54 |
| 5687 | 5103 | 89.73 | 11703 | 10256 | 87.64 |
|  | | **90.84±0.96** |  | | **89.49±1.61** |

Comparative efficiencies of the directed differentiation of MNs from the ALS patients’ hiPSCs.

**Table S5. The 106 PSC-CM protein candidates and their secretory capacity.**

| Name | Secreted | Non-secreted |
| --- | --- | --- |
| Aldolase C | o |  |
| Alpha Lactalbumin | o |  |
| ANGPTL3 | o |  |
| ApoA2 | o |  |
| ApoE | o |  |
| BCAM | o |  |
| MUC1 | o |  |
| MUC16 | o |  |
| Caspase-3 | o |  |
| Cathepsin B | o |  |
| CBP |  | o |
| TfR | o |  |
| CEA | o |  |
| Ceruloplasmin | o |  |
| Chemerin | o |  |
| CHI3L1 | o |  |
| CK-MB | o |  |
| Clusterin | o |  |
| C2 | o |  |
| C5a | o |  |
| Corticosteroid-binding globulin | o |  |
| C-Peptide | o |  |
| Troponin T | o |  |
| Cytokeratin 19 | o |  |
| BNP | o |  |
| ACTH | o |  |
| Exostosin-like 2 | o |  |
| Ferritin | o |  |
| Fibrinopeptide A | o |  |
| FSH | o |  |
| GLP-1 | o |  |
| GMNN |  | o |
| Hemopexin | o |  |
| HSP27 | o |  |
| HSP90 | o |  |
| IL-34 | o |  |
| Kallikrein 2 | o |  |
| Kallikrein 10 | o |  |
| Lyn | o |  |
| NPTXR |  | o |
| P-Cadherin | o |  |
| PIM2 |  | o |
| PPARg2 |  | o |
| PR Isoform B | o |  |
| PSA-free | o |  |
| PTPRD |  | o |
| Ret |  | o |
| Serpin A5 | o |  |
| SHBG | o |  |
| SOX2 |  | o |
| Angiogenin | o |  |
| Angiopoietin-1 | o |  |
| CCR5 |  | o |
| CD40 Ligand | o |  |
| Chordin-Like-1 | o |  |
| Cripto-1 | o |  |
| CXCR6 |  | o |
| EG-VEGF | o |  |
| ErbB3 | o |  |
| FGF-11 | o |  |
| FGF-19 | o |  |
| GDF-9 | o |  |
| GDF-11 | o |  |
| Granzyme A | o |  |
| GRO | o |  |
| HCR | o |  |
| NRG1 Isoform GGF2 | o |  |
| IFN-alpha/beta R2 | o |  |
| IGFBP-2 | o |  |
| IL-38 | o |  |
| IL-7 | o |  |
| IL-17 RB | o |  |
| IL-17 RD | o |  |
| IL-21 | o |  |
| IL-23 R | o |  |
| IL-26 | o |  |
| IL-29 | o |  |
| Insulysin |  | o |
| Kremen-2 |  | o |
| MFRP |  | o |
| MIF | o |  |
| MIP 2 | o |  |
| MMP-9 | o |  |
| MMP-16 | o |  |
| MMP-25 | o |  |
| NRG1 Isoform GGF2 | o |  |
| Orexin-A | o |  |
| Oncostatin M | o |  |
| OX40 Ligand | o |  |
| PDGF-AA | o |  |
| PDGF-C | o |  |
| ROBO4 |  | o |
| sFRP-1 | o |  |
| SIGIRR |  | o |
| SMAD1 | o |  |
| SMAD4 | o |  |
| SMAD5 | o |  |
| SMAD7 | o |  |
| Spinesin | o |  |
| TACI | o |  |
| Thrombospondin-4 | o |  |
| TIMP-1 | o |  |
| TLR1 |  | o |
| TRADD |  | o |
| TWEAK | o |  |
| VEGF-B | o |  |

Identity of the proteins that are upregulated in PCS-CM, as compared to the df-CM, and the secreted versa non-secreted properties of these proteins.
