# Supplementary material for: Autologous treatment for ALS with implication for broad neuroprotection
Source: Transl Neurodegener. 2022 Mar 11;11:16. doi: 10.1186/s40035-022-00290-5 (PMC8915496; doi:10.1186/s40035-022-00290-5)
Supplement: Supplementary file 2 — Additional file 2. Figure S1. Human embryonic stem cell-derived conditioned medium (hESC-CM) protects human fibroblast from H2O2 cytotoxicity. Figure S2. Human embryonic stem cell-derived conditioned medium (hESC-CM) protects motor neurons (MNs) from H2O2 cytotoxicity. Figure S3. Antioxidant activity is not different between human embryonic stem cell-derived conditioned medium (hESC-CM) and differentiated fibroblast-derived conditioned medium (dF-CM). Figure S4. Differentiation of ALS iPSCs into motor neurons (ALS-MNs). Figure S5. Profiling the time-point of the neuroprotective effect from iPSC-CM. Figure S6. The comparative effects of autologous ALS-derived hiPSC-conditioned media (ALS-CM) on neurites and apoptosis of ALS-motor neurons (ALS-MNs) in ALS patient-derived cell lines. Figure S7. The effect of CMs on the weight of SOD1G93A transgenic mice. Figure S8. The optimization of concentrations of heparin-binding protein (HBP) and exosome. Figure S9. The effect of epigenetic modifiers on the conditioned medium (CM). Figure S10. Differentially present proteins in each of the listed categories. [file 40035_2022_290_MOESM2_ESM.docx]

**Supplemental information: Figures and legends**

**
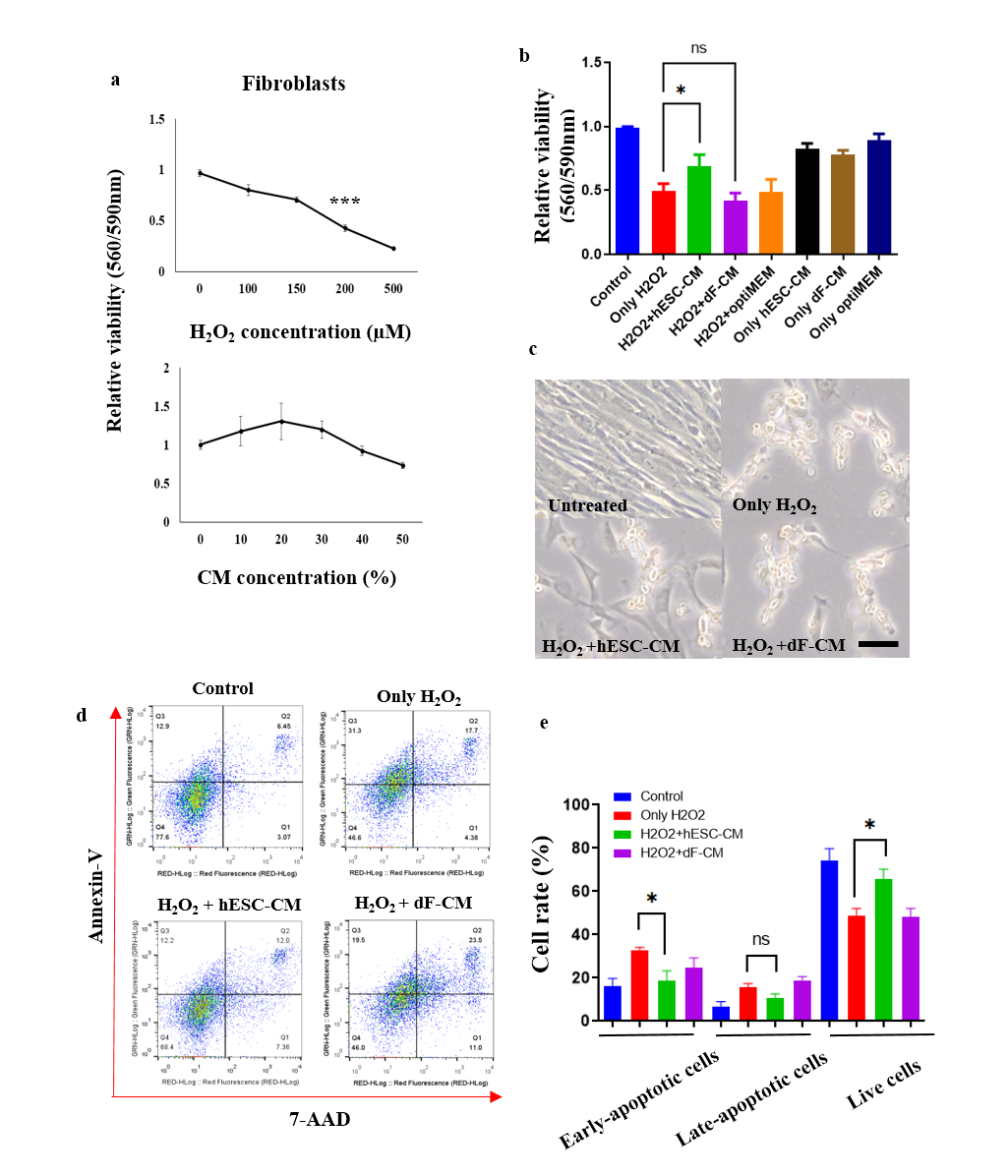
**

**Figure S1. Human embryonic stem cell-derived conditioned medium (hESC-CM) protects human fibroblast from H_2_O_2_ cytotoxicity.** **(a)** The viability of IMR90 fibroblasts was examined with different concentrations of H_2_O_2_ and with different concentrations of hESC-CM. Cell viability decreased significantly (below 0.5 value) by 200 μM H_2_O_2_ treatment. Viability was not affected by up to 30% hESC-CM. Data were normalized to the values at the 0 concentration. **(b)** MTT viability assay of IMR90 cells. Cell viability was noticeably lower in cultures treated with H_2_O_2_ and those co-treated with H2O2 plus the dF-CM as compared to the H_2_O_2_ plus hESC-CM cultures. The single treatment of each medium was presented as negative controls. **(c)** The morphologies of IMR90 cells that were cultured with/without H_2_O_2_, co-treated with hESC-CM or dF-CM. Scale bar, 100 μm. **(d)** Flow Cytometry dot-plot analyses of IMR90 cells that were treated with 200 μM H_2_O_2_ alone or co-treated with hESC-CM or dF-CM. As compared to the untreated control, the percent of apoptotic cells was much higher, and live cells significantly decreased with H_2_O_2_. Interestingly, the numbers of apoptotic cells robustly diminished and live cell numbers increased in cultures that were co-treated with H_2_O_2_ and hESC-CM, as compared to H_2_O_2_ alone or to H_2_O_2_ and dF-CM. **(e)** Quantification of Annexin V / 7AAD Flow Cytometry experiments. The numbers of apoptotic cells robustly diminished and live cell numbers increased in cultures that were co-treated with H_2_O_2_ and hESC-CM, as compared to H_2_O_2_ alone or to H_2_O_2_ and dF-CM. **P*<0.05, ****P*<0.001.


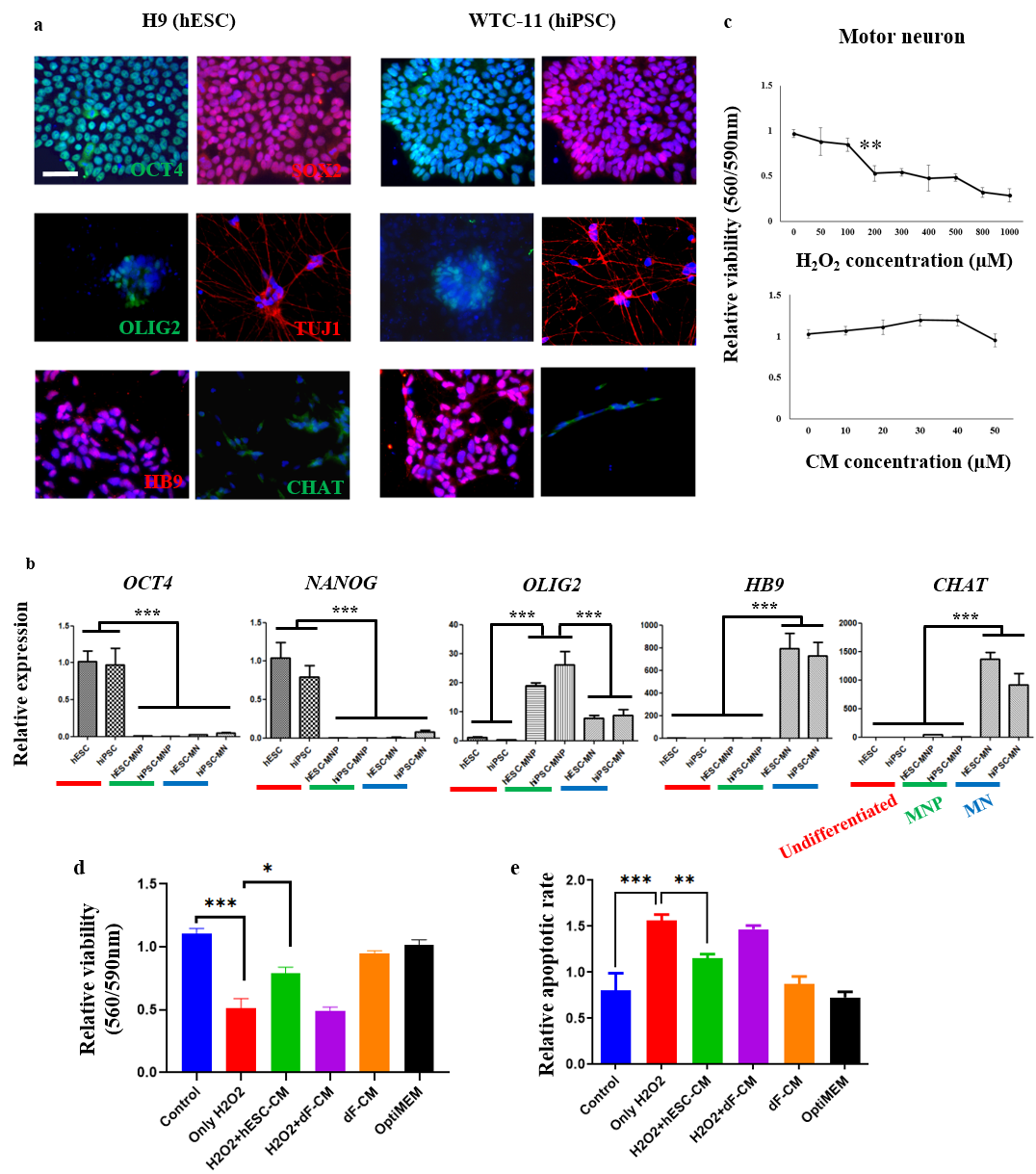


**Figure S2. Human embryonic stem cell-derived conditioned medium (hESC-CM) protects motor neurons (MNs) from H_2_O_2_ cytotoxicity. (a)** Differentiation of PSCs into MNs. The expression of stage specific markers for pluripotency (OCT4 and SOX2), motor neuron precursor (OLIG2), and MNs (TUJ1, BH9 and CHAT) by immunofluorescence; DAPI labels all nuclei. Scale bar, 100 μm. **(b)** Gene expression profiles of stage specific markers during differentiation into MNs by qRT-PCR that was performed in 3 replicates for each gene. ***P<0.001. After the motor-neuron identity and sufficient (above 90%) enrichment of these cultures were established, these human motor neurons were used in subsequent experiments. OLIG2 was detected at 14 days post differentiation. HB9, a specific marker for MNs, was observed at 30 days. Additionally, *HB9* and *CHAT* gene expression were expressed at 30 days and as expected, these were lacking in the undifferentiated PSCs. In concert, the expression of pluripotency-related genes decreased dramatically upon the directed differentiation into the motor neuron lineage. **(c)** The viability of MNs was examined with different concentrations of H_2_O_2_ and with different concentrations of hESC-CM. Motor neuron viability decreased significantly (below 50%) by 200 μM H_2_O_2_ treatment and was not changed by up to 40% hESC-CM**. (d)** MTT assay for viability of MNs that were cultured with 200 μM H_2_O_2_ and indicated media and CMs. **(e)** Annexin V apoptotic index of MNs exposed to 200 μM H_2_O_2_ in indicated culture conditions (fold of the first replicate of Control). When motor neurons were treated with H_2_O_2_ there was markedly diminished viability and increased apoptosis as compared to the untreated controls. However, the resilience of MNs to H_2_O_2_ cytotoxicity was significantly increased, by the hESC- CM. This contrasted with dF-CM that failed to improve viability of these MNs in the presence of H_2_O_2_. Annexin V fluorescence was assayed by a SpectraMax iD3 microplate reader. Bar graphs show Means and SEM of three independent experiments and subsequent triplicate assays (MTT, Flow cytometry, microplate reader of Annexin V). **P*<0.05, ***P*<0.01 ****P*<0.001.

**
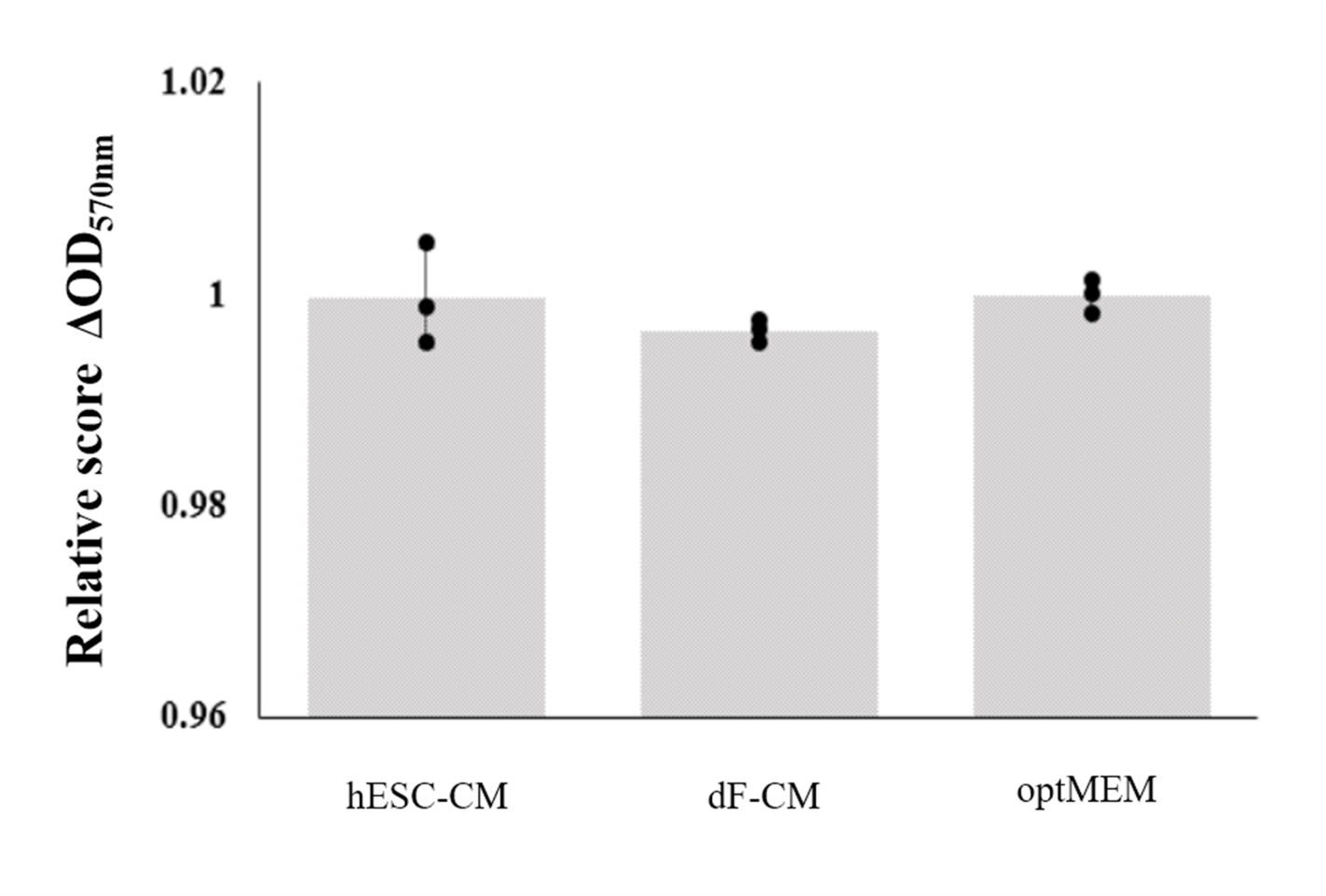
**

N. S.

**Figure S3. Antioxidant activity is not different between human embryonic stem cell-derived conditioned medium (hESC-CM) and differentiated fibroblast-derived conditioned medium (dF-CM).** Each CM was collected after culturing the respective cells in OptiMEM for 24 hours. Antioxidant activity assay (BioAssay systems, DTAC-100 kit) was performed in triplicate wells for each hESC-CM, dF-CM and the control medium, OptiMeM (optMEM), as recommended by the manufacturer. The antioxidant-reporting fluorescent signal intensity was measured by microplate reader (570 nm). The data are expressed as fold of the Mean of OptiMEM control, which was set at 1.


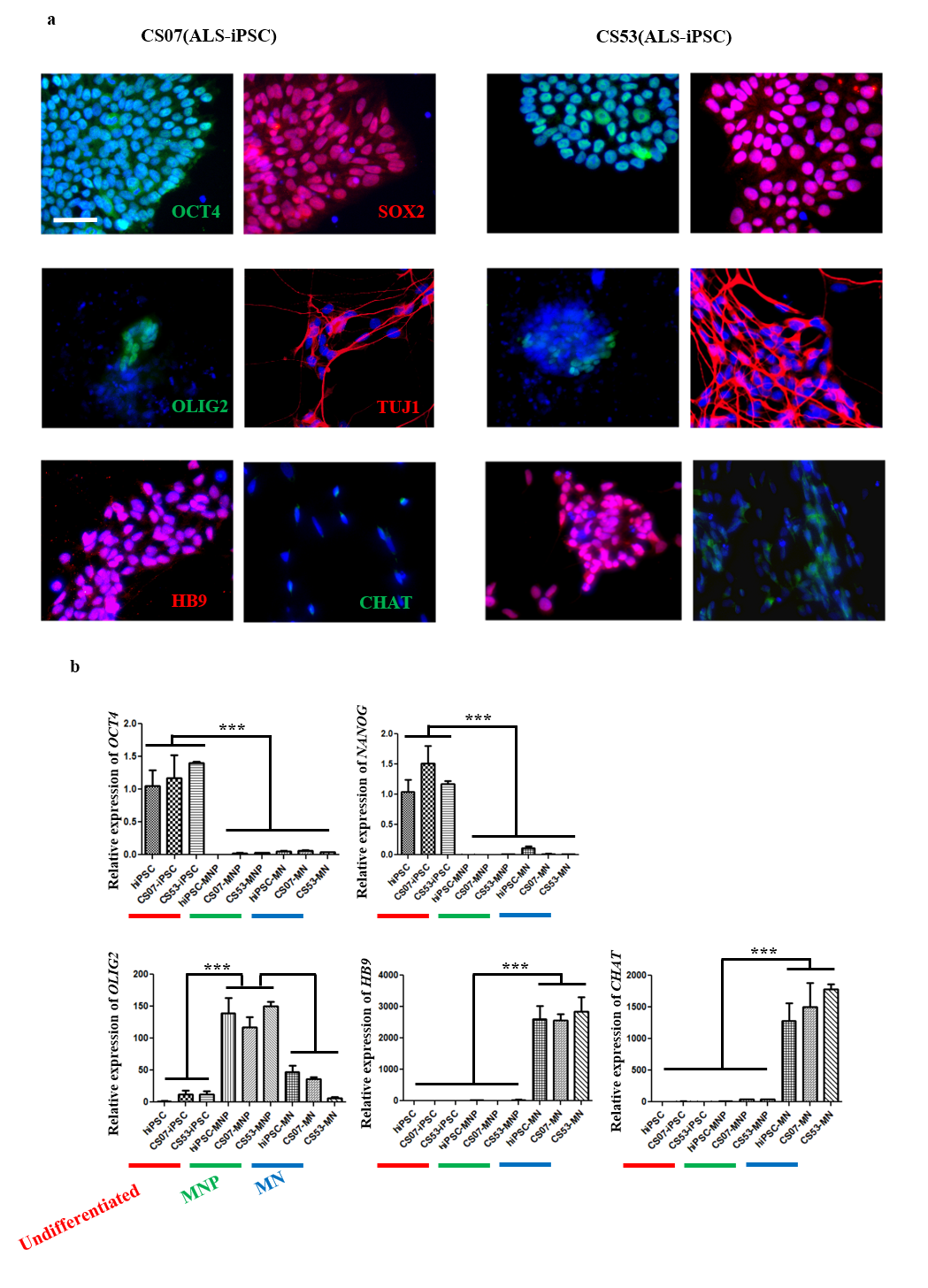


**Figure S4. Differentiation of ALS iPSCs into motor neurons (ALS-MNs**). **(a)** The expression of cell-fate markers for pluripotency (OCT4 and SOX2), motor neuron precursors (OLIG2), and MNs (TUJ1, BH9 and CHAT) by immunofluorescence; DAPI labels all nuclei. Scale bar, 100 μm. **(b)** qRT-PCR gene expression in 3 replicates for each gene assayed for the indicated cell-fate markers during differentiation of ALS-iPCSs into ALS-MNs ****P*<0.001.


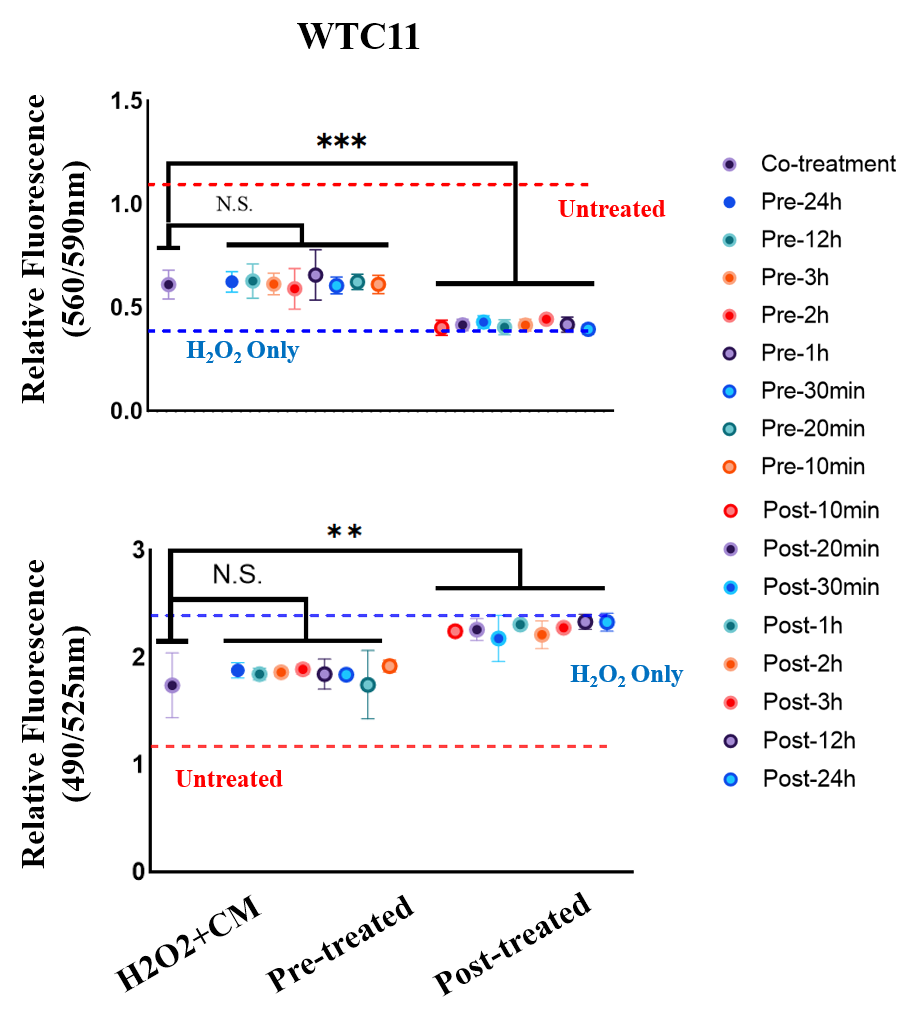


**Figure S5. Profiling the time-point of the neuroprotective effect from iPSC-CM.** The time course of viability (560/590nm) and apoptosis (490/590nm) of MNs that were pre-treated (before H2O2) or post-treated (after H_2_O_2_) with WT iPSC-CM.


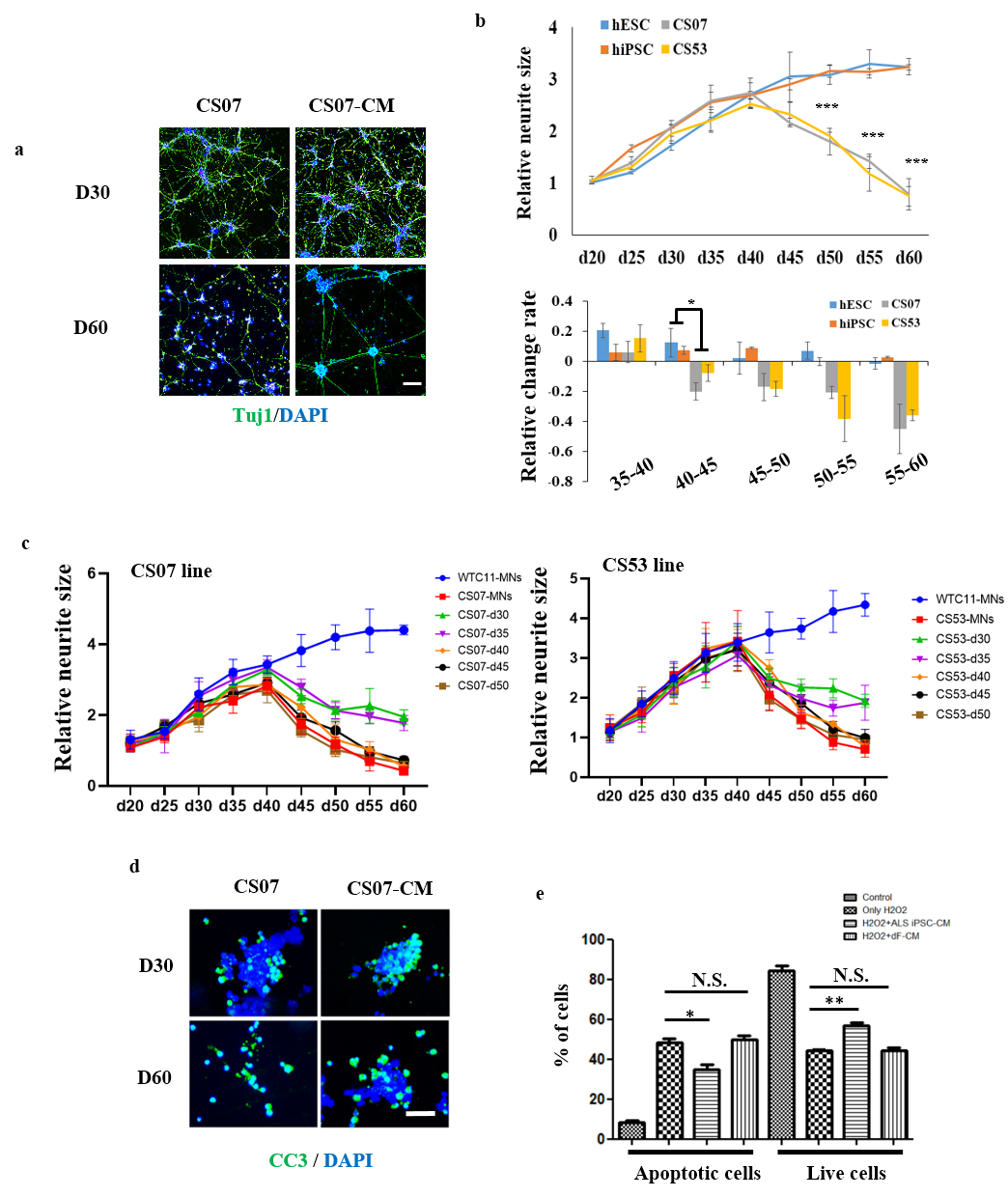


**Figure S6. The comparative effects of autologous ALS-derived hiPSC-conditioned media (ALS-CM) on neurites and apoptosis of ALS-motor neurons (ALS-MNs) in ALS patient-derived cell lines. (a)** Representative images of motor neurons with their neurites (CS07-ALS donor), (CS07-CM ALS donor iPSC conditioned medium) at 30 days and 60 days. Scale bar, 200 μm. **(b)** The neurite sizes of wild type and ALS-MNs were measured during cell differentiation (Upper). The net change of neurite size was calculated in these differentiating MNs (Below). **(c)** The neurite sizes were measured in ALS-MNs that were treated with autologous ALS-CM at different time points of their differentiation, d30, d35, d40, d45, and d50 (CS53, CS07). **(d)** Representative images of apoptotic Caspase 3 positive MNs at 30 days and 60 days of their directed differentiations of ALS iPSCs, CS07 line. Caspase 3 (green) was immunodetected with specific antibody, DAPI, blue stains all nuclei. Scale bar, 100 μm. **(e)** Quantification of apoptotic motor neuron precursors after H_2_O_2_ alone as compared to co-treatment with ALS iPSC-CM or dF-CM~~.~~ Values are mean ± SEM, *P<0.05, **P<0.01, N= triplicate assays for each condition.


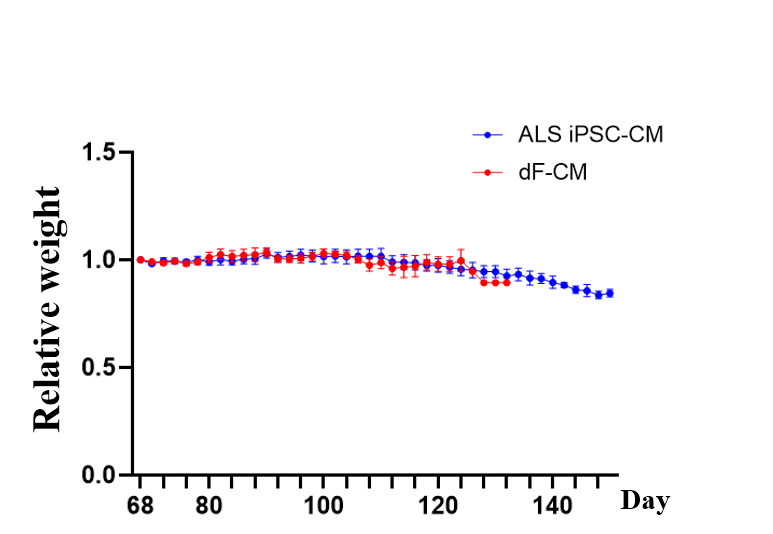


**Figure S7. The effect of CMs on the weight of SOD1^G93A^ transgenic mice.** The change of weights. There was no significant difference between ALS iPSC-CM group (*N*=16) and dF-CM group (*N*=6).


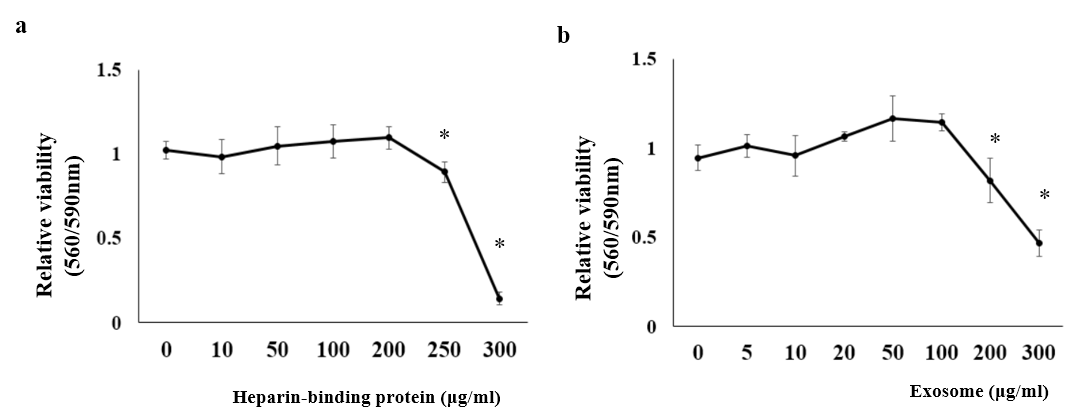


**Figure S8. The optimization of concentrations of heparin binding protein (HBP) and exosome. (a)** MTT assay for viability of MNs that were cultured with different concentrations of HBP fraction of hESC-CM. Quadruplicate assays for each condition. **(b)** MTT assay for viability of MNs that were cultured with different concentrations of exosome fraction of hESC-CM. Triplicate assays for each condition. **P*<0.05, Quadruplicates for each condition.

**
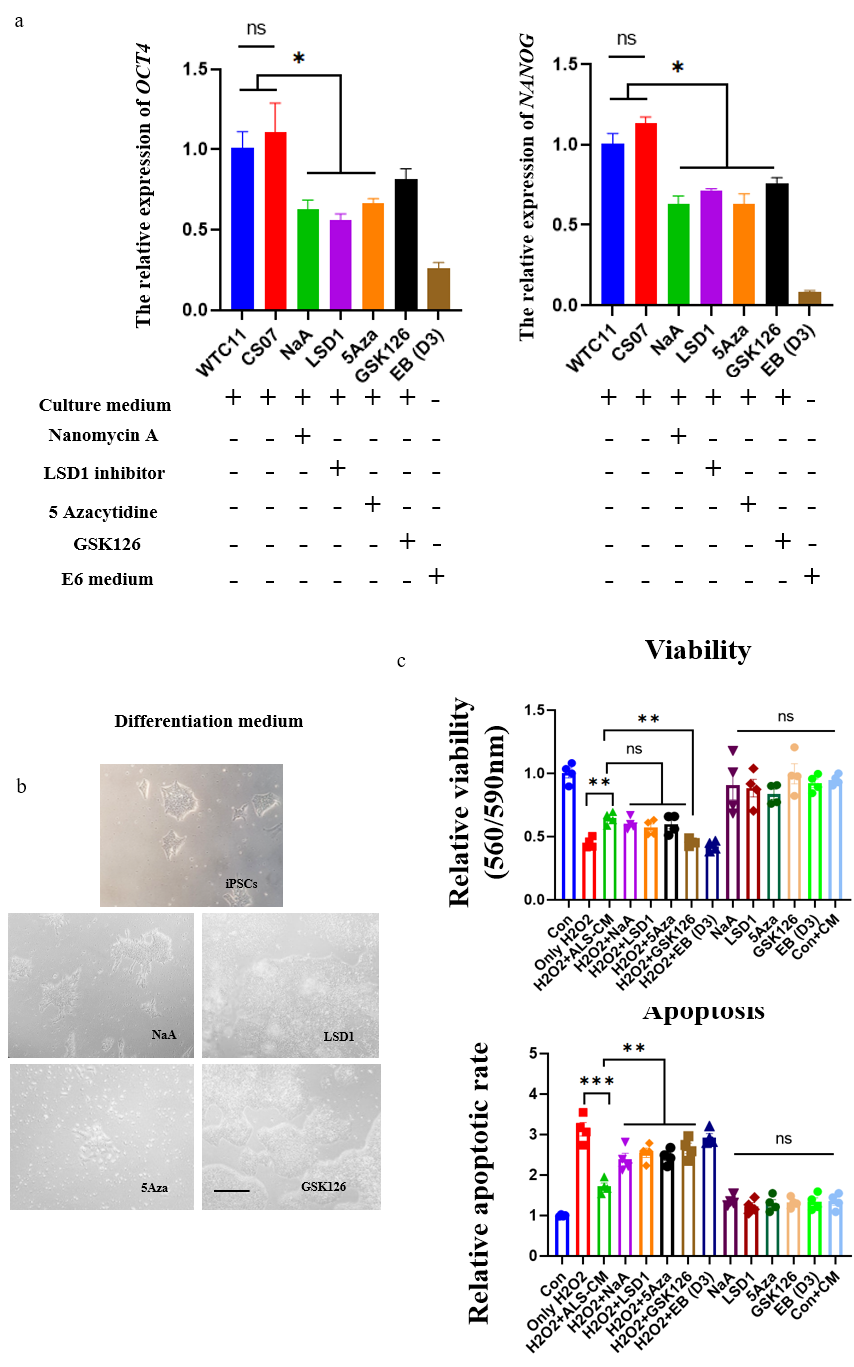
**

**Figure S9. The effect of epigenetic modifiers on the conditioned medium (CM). (a)** Gene expression profiles of pluripotency markers after treatment of four epigenetic modifiers, Nanomycin A (NaA), LSD1 inhibitor (LSD1), 5 Azacytidine (5Aza), and GSK126. The expression of *OCT4* was significantly decreased in the NaA, LSD1 and 5Aza groups compared to the untreated groups (WTC11 and CS07), but in the GSK126 group the decrease was not significant. *NANOG* expression significantly decreased in all groups treated with small molecules compared to the untreated groups. qRT-PCR was performed in 3 replicates for each gene. **(b)** The morphologies of induced pluripotent stem cells after the treatment of small molecules for 3 days. Scale bar, 200 μm. **(c)** The effect of epigenetic modifiers on the ability of CM to promote viability and inhibit apoptosis in ALS-derived MNPs. Bar graph represents Means and SDs of N=4 for each small molecule.


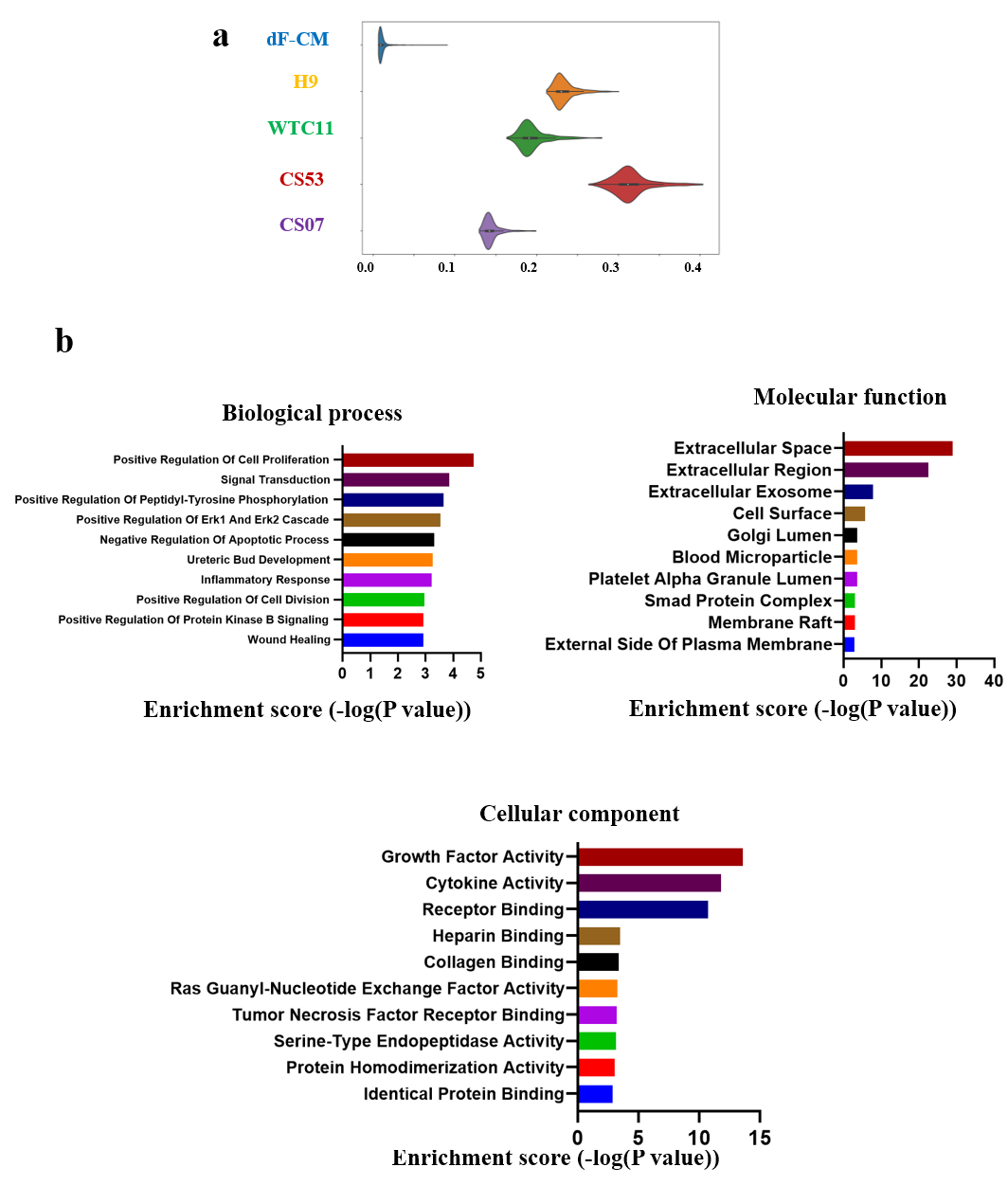


**Figure S10. Differentially present proteins in each of the listed categories. (a)** Violin plot of cohort distributions. **(b)** In the CC group, the top 10 most significantly up-regulated terms contained extracellular space, extracellular region, extracellular exosome, cell surface, blood microparticle, platelet alpha granule lumen, Golgi lumen, SMAD protein complex, membrane raft, and external side of plasma membrane. The top 10 most significantly up-regulated terms in the MF group comprised growth factor activity, cytokine activity, receptor binding, collagen binding, heparin binding, serine-type endopeptidase activity, tumor necrosis factor receptor binding, Ras guanyl-nucleotide exchange factor activity, protein homodimerization activity, and identical protein binding.
